# Glutathione Peroxidase-Like Activity of Amino-Substituted Water-Soluble Cyclic Selenides: A Shift of the Major Catalytic Cycle in Methanol

**DOI:** 10.3390/molecules22030354

**Published:** 2017-02-25

**Authors:** Kenta Arai, Ayako Tashiro, Yuui Osaka, Michio Iwaoka

**Affiliations:** Department of Chemistry, School of Science, Tokai University, Kitakaname, Hiratsuka-shi, Kanagawa 259-1292, Japan; yokoyaman45@yahoo.co.jp (A.T.); fairy_yui44@yahoo.co.jp (Y.O.)

**Keywords:** antioxidant, enzyme model, glutathione peroxidase, hydroxy perhydroxy selenane, selenide, selenoxide

## Abstract

We previously reported that water-soluble cyclic selenides can mimic the antioxidative function of glutathione peroxidase (GPx) in water through a simple catalytic cycle, in which the selenide (>Se) is oxidized by H_2_O_2_ to the selenoxide (>Se=O) and the selenoxide is reduced by a thiol back to the selenide. In methanol, however, the GPx-like activity could not be explained by this simple scenario. To look into the reasons for the unusual behaviors in methanol, monoamino-substituted cyclic selenides with a variable ring size were synthesized, and the intermediates of the catalytic cycle were characterized by means of ^77^Se-NMR and LC–MS spectroscopies. In water, it was confirmed that the selenide and the selenoxide mainly contribute to the antioxidative function, though a slight contribution from the dihydroxy selenane (>Se(OH)_2_) was also suggested. In methanol, on the other hand, other active species, such as hydroxyselenonium (>Se^+^–OH) and hydroxy perhydroxy selenane (>Se(OH)(OOH)), could be generated to build another catalytic cycle. This over-oxidation would be more feasible for amino-substituted cyclic selenides, probably because the ammonium (NH_3_^+^) group would transfer a proton to the selenoxide moiety to produce a hydroxyselenonium species in the absence of an additional proton source. Thus, a shift of the major catalytic cycle in methanol would make the GPx-like antioxidative function of selenides perplexing.

## 1. Introduction

**Scheme 1:**
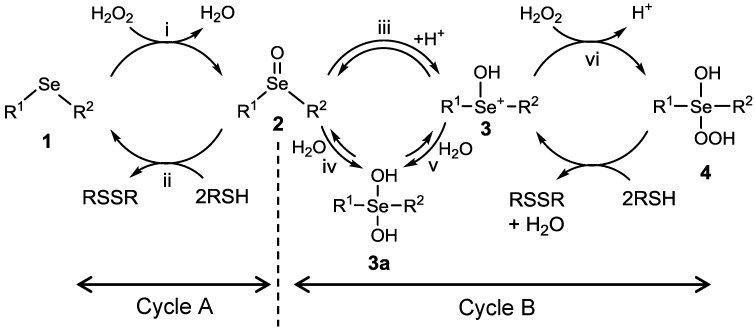
Proposed glutathione peroxidase (GPx)-like catalytic cycle of a selenide. (**A**) selenide/selenoxide redox pathways mainly observed in water [26,27,28,29]; (**B**) another catalytic cycle mediated by hydroxyselenonium **3** and hydroxy perhydroxy selenane **4** as a highly active oxidant [32].

Reactive oxygen species (ROS)—such as hydrogen peroxide (H_2_O_2_), superoxide anion (·O^2−^), hydroxyl radical (HO·), and singlet oxygen—which are steadily generated during the consumption processes of triplet oxygen, have high oxidizability to damage DNAs, cellular lipids, and proteins, leading various life-threatening disorders, such as protein-misfolding disease in central nervous systems, myocardial infarction in heart, diabetes in liver, and cataract in eyes [1]. To protect themselves from such oxidative stress, eukaryotic organisms have evolved systems involving various antioxidative enzymes. Glutathione peroxidase, a representative antioxidative enzyme and a well-known selenoenzyme, has selenocysteine (Sec), a selenium analog of natural cysteine, at the active site. Glutathione peroxidase (GPx) catalyzes the reduction of H_2_O_2_ to harmless water (H_2_O) using glutathione (GSH) as a reducing cofactor. From a view point of drug design for antioxidant therapy, much effort has been directed for decades toward development of organoselenium compounds [2,3,4,5,6,7], which can act as GPx-like catalysts, and also toward elucidation of their catalytic mechanisms [8,9,10,11]. Various GPx model compounds, most of which are aromatic diselenides (ArSeSeAr), have been developed and their catalytic activities have been evaluated [12,13,14,15,16,17,18,19,20,21,22,23,24,25]. In the meantime, aliphatic and/or aromatic monoselenides (RSeR′) are also known to act as GPx-like catalysts through a unique selenide/selenoxide redox cycle (Scheme 1, cycle A), in which selenide **1** is oxidized by H_2_O_2_ to the corresponding selenoxide **2**, which is reduced back to **1** by a thiol substrate (RSH) [26,27,28,29]. In addition, Back et al. reported that bis(3-hydroxypropyl) selenide can be oxidized to a reactive spiro dioxoselenurane species, instead of a selenoxide, which is also reduced back to the corresponding selenide by thiols [30,31]. Recently, Braga and coworkers demonstrated the presence of another catalytic cycle (cycle B) [32]. According to their report, selenoxide **2** is in equilibrium with dihydroxy selenane **3a** or hydroxyselenonium **3** in methanol, and **3** reacts with H_2_O_2_ to generate a highly reactive species (i.e., hydroxy perhydroxy selenane **4**), which is a much greater oxidant than **2** for thiol substrates.

In our laboratory, a series of water-soluble cyclic selenides, such as **7**‒**9**, have been synthesized, and their potential GPx-like activities were evaluated in water as well as in methanol [26,27,29]. Our previous analysis revealed that they exhibit the activity through a simple redox interconversion between a selenide and a selenoxide (i.e., cycle A in Scheme 1) in an aqueous medium, and also that the magnitude of the activity can be controlled by changing the ring size and the polar functional groups attached to the ring. The optimal ring size was five, and the GPx-like catalytic activity generally became lower when the substituent on the ring structure was changed from carboxy (CO_2_H) to hydroxy (OH), and then amino (NH_2_) groups. Since CO_2_H should exist as an ionized form (CO_2_^−^), this substituent would facilitate the conversion of selenide to selenoxide (reaction i in Scheme 1)—which is a rate-limiting step in the catalytic cycle A—by elevating the HOMO (highest occupied molecular orbital) energy level of the selenide by inductive electron releasing. On the other hand, a protonated amino group (NH_3_^+^) should decelerate the reaction due to the inductive electron withdrawal. Although a magnitude of GPx activity of the cyclic selenides correlates well with both the HOMO energy level and the second-order rate constant for the oxidation of selenide to selenoxide (reaction i) in water, cyclic selenide **7**, with a five-membered ring having one amino substituent, showed unexpectedly high activity [29].

In methanol, on the other hand, cyclic selenides having amino substituents exhibited higher GPx-like activity than those having OH or CO_2_H substituents. Thus, the activity rank of cyclic selenides could not be explained by the simple scenarios, which were applicable in water [29], suggesting that the presence of other catalytic cycles, such as cycle B. In this context, it would be important to assess an effective GPx-like catalytic cycle in methanol for cyclic selenides, especially for those having amino substituents, in order to explain the unusual behaviors.

**Figure 1:**
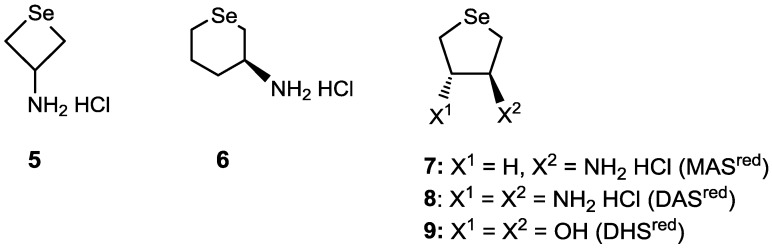
Target compounds in this study (**5** and **6**) and previous selenides (**7**‒**9**). Compounds **8** and **9** are racemic compounds.

In this study, monoamino-substituted cyclic selenides with a four- (**5**) or six-membered ring (**6**) were additionally synthesized, and their GPx-like catalytic activity and the catalytic cycle were investigated in water and in methanol by using monoamino cyclic selenide **7** (MAS^red^), diamino cyclic selenide **8** (DAS^red^), and dihydroxy cyclic selenide **9** (DHS^red^) as reference selenides. The selenide compounds employed in this study are lined up in Figure 1.

## 2. Results and Discussion

### 2.1. Synthesis

**Scheme 2:**
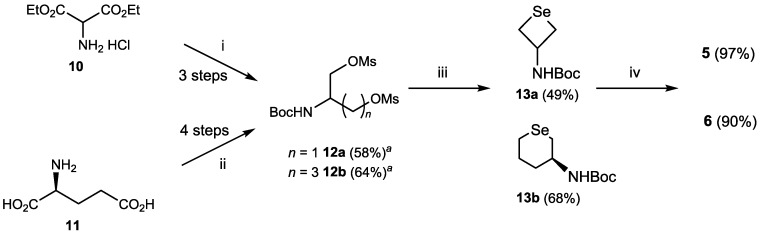
Synthesis of monoamino selenides **5** and **6**. *Reagents and conditions*: (i) (1) Boc_2_O, Et_3_N, 1,4-dioxane/H_2_O (5:2), 50 °C, 18 h, (2) NaBH_4_, EtOH, reflux, 1 h, (3) MsCl, Et_3_N, CH_2_Cl_2_, 20 h, room temperature (rt); (ii) (1) EtOH, AcCl, reflux, 4 h, (2) Boc_2_O, Et_3_N, 1,4-dioxane:H_2_O = 5:2, 50 °C, 18 h, (3) NaBH_4_, EtOH, reflux, 1 h, (4) MsCl, Et_3_N, CH_2_Cl_2_, 20 h, rt; (iii) NaHSe, *i*PrOH/1,4-dioxane, reflux, 2.5 h for synthesis of **13a** from **12a**; NaHSe, EtOH/THF, reflux, 3 h for synthesis of **13b** from **12b**; (iv) HCl, H_2_O/EtOH, 35 °C, 20 h. ^a^ Details of the synthesis are given in Supporting Information.

Compounds **5** and **6** were synthesized by applying a similar protocol reported previously [33] (Scheme 2). Boc-protected mesylates **12a** and **12b** were first prepared from diethyl aminomalonate hydrochloride (**10**) or l-glutamic acid (**11**), respectively, by following previous methods with slight modifications [34,35]. The mesylates were then converted into cyclic selenides **13a** and **13b** by selenation with NaHSe. Finally, obtained **13a** and **13b** were treated with HCl to give target compounds **5** and **6**, which were fully characterized by ^1^H, ^13^C, and ^77^Se-NMR as well as high-resolution mass spectroscopy (HRMS).

### 2.2. Redox Properties of Selenides and Selenoxides in Water

The redox reactions of synthesized **5** and **6** were monitored by ^77^Se-NMR spectroscopy in D_2_O. The spectral changes observed for **6** are shown in Figure S1 as a typical example. When **6** was reacted with 1 equivalent of H_2_O_2_, the broad peak of **6** (136 ppm) completely disappeared after 2 h, and two signals (829 ppm (major) and 834 ppm (minor)), which should correspond to two stereoisomers of the selenoxide, appeared (Figure S1b). Quantum chemical calculation at B3LYP/6-31+G(d,p) showed that the *cis*-isomer, which has an axial amino group on the six-membered ring, is 6.02 kcal/mol more stable than the *trans*-isomer, which has an equatorial amino group, in implicit water. Thus, the major peak (829 ppm) in the NMR spectrum was assigned to the *cis*-isomer, in which a hydrogen atom of the protonated ammonium (NH_3_^+^) group would form an intramolecular hydrogen bond with the oxygen atom of the selenoxide moiety. The selenoxide was reduced back to selenide **6** within 15 min after addition of 1 equivalent of dithiothreitol (DTT^red^). For five-membered cyclic selenide **7**, similar spectral changes were observed in the ^77^Se-NMR analysis (Figure S3). Quantum chemical calculation again suggested that among the two stereoisomers of the selenoxide the *cis*-isomer is more stable.

According to Scheme 1, selenoxide **2** can be further oxidized by an excess amount of H_2_O_2_ to hydroxy perhydroxy selenane **4** through hydroxyselenonium **3** and/or dihydroxy selenane **3a** (cycle B). To confirm whether such over-oxidation is possible or not, the selenoxide obtained from **6** (Figure S1b) was reacted with 4 equivalents of H_2_O_2_. However, obvious spectral changes were not observed (Figure S1c). Nevertheless, when 4 equivalents HCl were further added as a proton source, the major selenoxide signal disappeared on the NMR chart, leaving the minor *trans*-isomer with an equatorial ammonium (NH_3_^+^) group unreacted (Figure S1d). At this stage, any new signals were not detected over a range of 0–1500 ppm in the ^77^Se-NMR spectrum, probably because of significant line broadening of the absorption peak for the generated species, which would be the corresponding hydroxyselenonium **3** or dihydroxy selenane **3a**. Preferential conversion of the *cis*-isomer suggests that the protonated axial NH_3_^+^ group can assist conversion of the selenoxide by interacting with the selenoxide oxygen, whereas the equatorial NH_3_^+^ group cannot assist it. It should be noted that the minor isomer of the selenoxide was not reacted, even when 6 equivalents in total of HCl was added. When the resulting solution (Figure S1d) was added with 5 equivalents of DTT^red^, the selenide **6** was regenerated, suggesting that the tetrahydroselenopyran skeleton was not decomposed during the conversion to active species. In addition, when the solution of the selenoxide (Figure S1b) was added with 4 equivalents HCl in the absence of H_2_O_2_, only the major peak of the selenoxide disappeared, probably due to its conversion into hydroxyselenonium **3** or dihydroxy selenane **3a** (Figure S1e). The selenoxide was recovered by neutralization with 4 equivalents of NaOH (Figure S1f), suggesting that selenoxide **2** and hydroxyselenonium **3** are in equilibrium with each other (reaction iii in Scheme 1). Similar redox behaviors were observed for other amino selenides **5**, **7**, and **9** (Figures S2–S5), although the selenoxide of **5** was decomposed by addition of an excess amount of H_2_O_2_ and HCl (Figure S2). For the selenoxide of DHS^red^ (**9**), a new signal (959 ppm), which would correspond to **3** or **3a**, appeared with an accompanying disappearance of the selenoxide peak (926 ppm) when 4 equivalents of HCl was added to the solution of the selenoxide (Figures S5 and S6). The signal at 959 ppm disappeared and the selenoxide signal appeared after neutralization with 4 equivalents of NaOH (Figure S6).

**Figure 2:**
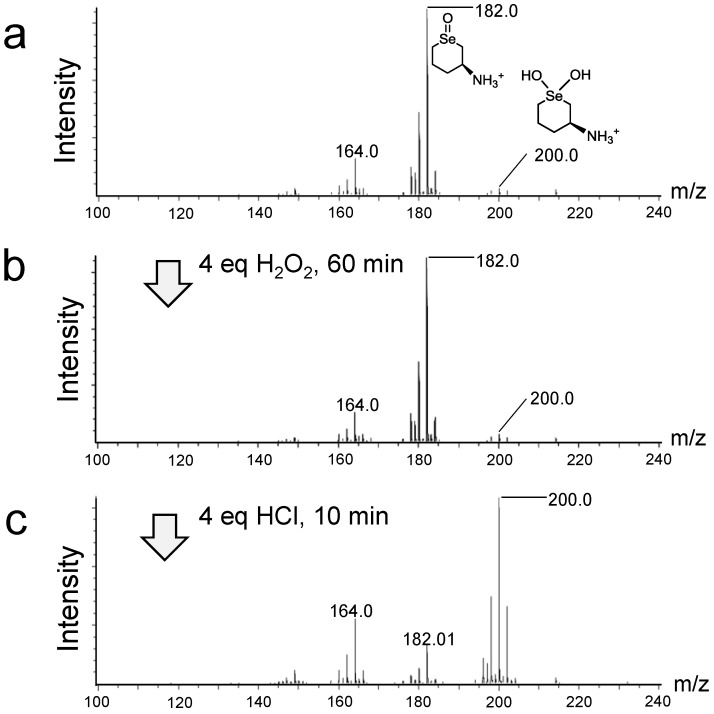
LC–MS (atmospheric-pressure chemical ionization, APCI+) spectral changes during the oxidation of the selenoxide form of selenide **6** in H_2_O at 25 °C. For **a**‒**c**, H_2_O (100%) was used as an eluent for the LC under a continuous flow at 0.3 mL/min, and 3 μL of the sample solution was injected into the LC and analyzed by the APCI+ mode. *Reaction conditions*: (**a**) Selenide **6** (0.038 mmol) and H_2_O_2_ (0.038 mmol) were mixed in H_2_O (800 μL); (**b**) to **a** was added H_2_O_2_ (0.15 mmol); (**c**): to **b** was added HCl (0.15 mmol).

In order to identify the products of the above reactions, the sample solution during the redox reaction of selenides with H_2_O_2_ was analyzed by LC–MS (atmospheric-pressure chemical ionization (APCI) and electrospray ionization (ESI)) in a positive ion mode under a continuous flow condition (0.3 mL/min). Typical MS spectra obtained for **6** are shown in Figure 2. After treatment of the selenide with 1 equivalent of H_2_O_2_ in water for 2 h, the selenoxide (C_5_H_12_NO^80^Se^+^) was observed as a major product, which was detected at *m*/*z* 182.0 (Figure 2a), while the small peak of dihydroxy selenane **3a** (C_5_H_14_NO_2_^80^Se^+^), was detected at *m*/*z* 200.0. Further addition of H_2_O_2_ (4 equivalents) did not cause dramatic changes in the MS spectrum (Figure 2b). However, when 4 equivalents of HCl were added, the peaks of the selenoxide converted into **3a** (Figure 2c), which would be generated through reaction iii to v or reaction iv in Scheme 1. Furthermore, under an ESI+ condition, hydroxy perhydroxy selenane **4** (C_5_H_14_NO_3_Se^+^) was slightly but obviously detected (Figure S7), supporting that the selenoxide can be over-oxidized to **4** under an acidic condition via hydroxyselenonium **3**. Very recently, based on theoretical calculation, Orian et al. reported that the conversions of a selenoxide into the corresponding dihydroxy selenane and hydroxy perhydroxy selenane are significantly endothermic (+13.1 and +16.4 kcal/mol, respectively) [36]. This is consistent with our observation that the conversion of selenoxide **2** into **3a** and **4** was not easy unless HCl and/or excess H_2_O_2_ were added. When the same experiments were performed for monoamino selenide **7**, similar results were obtained (Figure S8). For selenide **5**, on the other hand, the reaction with H_2_O_2_ produced significant amounts of decomposed compounds, for which the structures could not be characterized.

The results of LC–MS analysis strongly indicate that the simple redox reaction between selenide and selenoxide (i.e., reaction i and ii in Scheme 1) is predominant during the GPx-like catalytic reaction under a physiological pH condition (i.e., pH 7.4), while a small portion of the selenoxide, when it has an amino substitution, could be over-oxidized.

### 2.3. GPx-Like Activity of Selenides ***5**‒**9*** at pH 7.4

**Figure 3:**
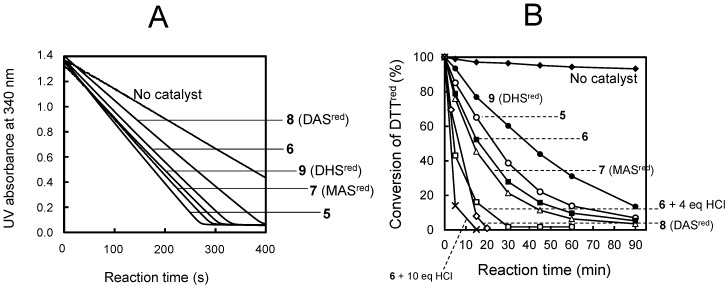
GPx-like activity assay in buffer solution and in methanol. (**A**) Nicotinamide adenine dinucleotide phosphate (NADPH)-coupled GPx assay for selenides **5**–**9**. Reaction conditions were [GSH]_0_ = 1.0 mM, [H_2_O_2_]_0_ = 2.5 mM, [NADPH]_0_ = 0.3 mM, [glutathione reductase] = 4 units/mL, and [selenide] = 0.2 mM in pH 7.4 phosphate buffer at 25 °C. (**B**) Percentages of residual dithiothreitol (DTT^red^) as a function of reaction time in the oxidation of DTT^red^ with H_2_O_2_ in the presence of a selenide catalyst (**5–9**) in CD_3_OD. Reaction conditions were [DTT^red^]_0_ = [H_2_O_2_]_0_ = 0.14 M and [selenide] = 0.014 M at 25 °C. Data for **7**‒**9** were quoted from Reference [29].

The GPx-like catalytic activities of the selenides were evaluated in a buffer solution at pH 7.4 in the nicotinamide adenine dinucleotide phosphate (NADPH)-coupled assay [27,37]. In this assay, the decomposition of H_2_O_2_ was monitored by NADPH consumption at 340 nm. The results are graphically shown in Figure 3A. The initial velocities (ν_0_) of H_2_O_2_ decomposition catalyzed by various selenides are summarized in Table 1.

Since the rate-determining step of cycle A is the oxidation step (reaction i), GPx-like catalytic activity of selenides in water at pH 7.4 should depend on the second-order rate constants (*k*_ox_) for the reaction between the selenide and H_2_O_2_. Following the previous protocol [29], the *k*_ox_ value was determined for **6** (Table 1). However, the *k*_ox_ value for selenide **5** could not be determined due to the decomposition as mentioned above. The order of *k*_ox_ and ν_0_ values obtained for amino selenides were **7** > **6** > **8** and **5** > **7** > **6** > **8**, respectively, which were in complete agreement with each other. The order was also consonant with the order of the HOMO energy levels calculated in water (Table 1), although diamino selenide **8** had a much higher HOMO level, probably due to the presence of two NH_3_^+^ substituents.

### 2.4. GPx-Like Activity Assay in Methanol

The GPx-like activities were evaluated by the NMR method as described previously [26,27]. Oxidation of dithiothreitol (DTT^red^) was initiated by addition of H_2_O_2_ to a mixture of DTT^red^ and a catalytic amount (10 mol %) of a selenide in CD_3_OD. The reaction was carried out in a NMR sample tube at 298 K. The ^1^H-NMR spectrum of the solution was measured after a certain period of time. The change of the relative signal intensities of DTT^red^ (δ = 3.67, 2H) and DTT^ox^ (δ = 3.03, 2H) was integrated to calculate the percent conversion of DTT^red^ to DTT^ox^ (Figure 3B). The times for 50% conversion (*t*_50_) obtained are summarized in Table 1. All selenides catalyzed the oxidation of DTT^red^ with H_2_O_2_. The order of the catalytic activity (i.e., the inverse of *t*_50_) was **8** > **7** > **6** > **5** > **9**, which did not match with the order in water (i.e., the magnitude of ν_0_), **5** > **7** > **9** > **6** > **8**. In addition, it is obvious that the amino groups on the rings had a proclivity to enhance the activity in methanol. Similar results had been observed in our previous study [29]. These observations suggest that the reaction should be catalyzed through not only cycle A, but also through another cycle, which would involve hydroxy perhydroxy selenane **4** (cycle B). This assumption was indeed supported by the following analyses.

### 2.5. Components of the Reaction Solution in Methanol

**Figure 4:**
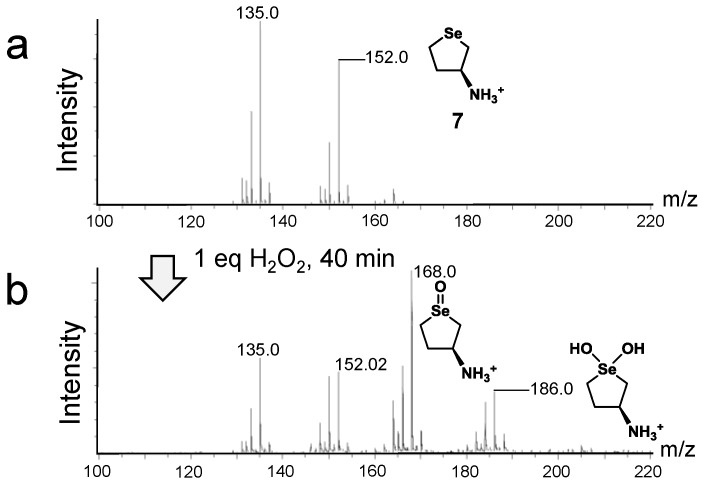
LC–MS (APCI+) spectral changes during the redox reactions of **7** in MeOH at 25 °C. MeOH (100%) was used as an eluent for the LC. *Reaction conditions*: (**a**) selenide **7** (0.038 mmol) in MeOH (800 μL); (**b**) to **a** was added H_2_O_2_ (0.038 mmol).

To obtain information about a shift of the catalytic cycle in methanol, the components of the reaction mixture were analyzed by means of ^77^Se-NMR and LC–MS (APCI+ or APCI−) spectroscopies. The results of NMR analysis obtained for selenide **7** are shown in Figure S9. ^77^Se-NMR spectra in CD_3_OD showed that **7** (δ = 148 ppm, Figure S9a) was oxidized by 1 equivalent of H_2_O_2_ to the selenoxide (δ = 967 ppm (major) and 949 ppm (minor), Figure S9b). The two peaks can be assigned to *cis*- and *trans*-stereoisomers, respectively, because ab initio calculation showed that the *cis*-isomer, which forms an intramolecular hydrogen bond, is 6.24 kcal/mol more stable than the *trans*-isomer. When 1 equivalent of DTT^red^ was added, the selenoxide was rapidly reduced to selenide **7**. On the other hand, when 4 equivalents H_2_O_2_ were added to the selenoxide, the major isomer (δ = 968 ppm) preferentially converted into other species (Figure S9c). This is in significant contrast to the reaction in water, where the selenoxide was unreacted in the absence of HCl. The selenoxide was completely converted into other species, for which no signal was detected over the range of 0–1500 ppm, by further addition of 4 equivalents HCl (Figure S9d). When 5 equivalents of DTT^red^ were added to the resulting sample solution, the selenide was regenerated, suggesting that the selenoxide did not decompose but was converted into active species. In addition, when the solution of the selenoxide (Figure S9b) was added with 4 equivalent of HCl, the selenoxide completely reacted, probably to produce hydroxyselenonium **3** or dihydroxy selenane **3a**, as observed for **6** in aqueous solution (Figure S9e). By neutralization with NaOH, the selenoxide was recovered (Figure S9f). On the other hand, the LC–MS (APCI+) analysis showed that by reacting selenide **7** (Figure 4a) with 1 equivalent of H_2_O_2_, generation of a considerable amount of dihydroxy selenane **3a** (*m*/*z* 186.0; C_4_H_12_O_2_^80^Se^+^) was observed along with the corresponding selenoxide (*m*/*z* 168.0; C_4_H_10_NO^80^Se^+^) (Figure 4b), suggesting that **3a** was in equilibrium with selenoxide **2** in methanol although HCl was absent. When four equivalents of H_2_O_2_ were further added to the resulting solution, small signals were observed at around *m*/*z* 202.0, which corresponded to species **4** (C_4_H_12_NO_3_^80^Se^+^) (Figure S10). Similar redox behaviors were also observed for selenide **6**, although the generation of the corresponding dihydroxy selenane **3a** was less than that of selenide **7** when **6** was reacted with excess H_2_O_2_ (Figure S11). On the other hand, oxidation of selenide **5** by one equivalent of H_2_O_2_ in methanol provided complicated MS spectra, showing the formation of not only selenoxide and species **3a** but also several decomposed species. For **9**, NMR analysis showed that conversion of the selenoxide to an active species was progressed when excess H_2_O_2_ and HCl were both added (Figure S12).

**Scheme 3:**
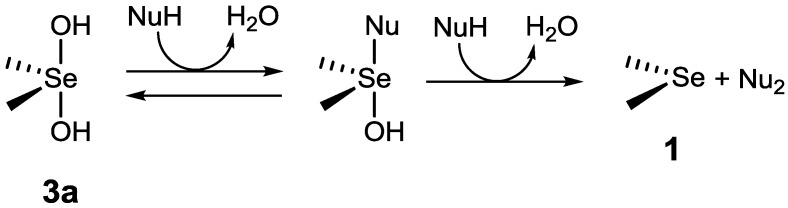
Oxidation of a nucleophile (NuH) by dihydroxyselenane **3a**.

The NMR and LC–MS analyses suggested that in methanol amino-substituted selenides could be over-oxidized to **4** by excess amounts of H_2_O_2_ even in the absence of HCl. It was, therefore, assumed that in addition to cycle A of Scheme 1, cycle B would work in methanol for monoamino selenides, except for **5**. It is known that dihydroxy selenane **3a** acts as a good oxidant for nucleophilic substrates through the exchange of the hydroxy ligands, as shown in Scheme 3 [38,39,40,41]. Dihydroxy tellurane, which is a tellurium analog of **3a**, can also oxidize thiols to disulfides through similar ligand exchange [42]. Thus, the **3a** could be involved in the catalytic reaction as an active intermediate via the cycle other than cycles A and B.

### 2.6. Determinant Factors of the GPx-Like Activity of Selenides

**Scheme 4:**
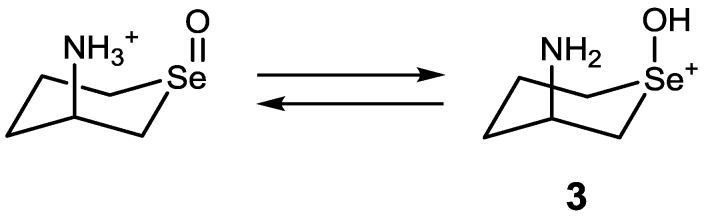
A proposed equilibrium between an amino-substituted cyclic selenoxide and the corresponding hydroxyselenonium **3**.

According to component analysis during the redox reaction in methanol, it appeared that in methanol convertibility of selenoxides to **3**, **3a**, and **4** would correlate to the GPx-like activity. Indeed, the catalytic reaction of selenide **6** in methanol was dramatically accelerated when 4 or 10 equivalents of HCl were added to the reaction solution, probably due to the enhancement of convertibility of **6** into such active species (Figure 3B). It should be noted that the reaction was not accelerated when HCl was added to the blank solution in the absence of a selenide catalyst. For the series of monoamino-substituted selenides, it was suggested that over-oxidation of the selenoxide to **4** can be progressed by an excess amount of H_2_O_2_ even in the absence of a proton source (Figure 4, Figures S9 and S11). This is probably because the *cis*-isomer of the selenoxide having a protonated ammonium (-NH_3_^+^) group in an axial direction is in equilibrium with hydroxyselenonium **3** as shown in Scheme 4. Such equilibrium, however, cannot occur for the selenoxide of hydroxy-substituted selenide **9**. A preferential conversion of the *cis*-isomer as observed in Figures S1 and S9 would support the presence of the equilibrium of Scheme 4.

In aqueous medium, on the other hand, generation of species **3** was not obviously observed for any selenides in the absence of HCl. This is probably because hydration around the selenoxides inhibits the proton transfer from NH_3_^+^ to oxygen atom to form **3**, which might further react with H_2_O_2_ to form **4**. Indeed, distances of the intramolecular hydrogen bonds between NH_3_^+^ and the selenoxide moiety calculated for selenides **6** and **7** were ca. 0.01 Å longer in water than those in methanol, supporting more difficult conversion of the selenoxide to **3** in water than in methanol. Thus, the strength of the hydrogen bonding to the selenoxide moiety may be one of the factors to determine the convertibility of cyclic selenoxides into other active species, such as **3**, **3a** and **4**, and hence enhance the GPx-like activity in methanol. It should be noted though that the calculated hydrogen bond distance of the selenoxide of **7** was slightly longer than that of the selenoxide of **6**, the catalytic activity of **7** was larger than that of **6**.

## 3. Material and Methods

### 3.1. General

^1^H (500 MHz), ^13^C (125.8 MHz), and ^77^Se (95.4 MHz) NMR spectra were recorded at 298 K, and coupling constants (*J*) are reported in hertz. High-resolution mass spectra (HRMS) and low-resolution mass spectra (MS) were recorded under atmospheric-pressure chemical ionization (APCI+ or APCI−) or electrospray ionization (ESI+) conditions. All reactions for the synthesis of selenides were monitored by thin-layer chromatography (TLC). Gel permeation chromatography (GPC) was performed with a general isocratic HPLC system using CHCl_3_ as the eluent. Ultraviolet (UV) spectra were measured at 25.0 °C using a circulating water-bath system. Selenides **7** [33], **8** [29], and **9 [43]** were prepared as described. All other chemicals were used as purchased without further purification. NADPH-coupled GPx activity assay in buffer solution at pH 7.4, GPx-like activity assay by NMR in CD_3_OD, and kinetic analysis for selenide oxidation with hydrogen peroxide in water were performed by following our previous literatures [26,27,29].

*3-(tert-Butoxycarbonylamino)selenetane* (**13a**). Selenium powder (0.289 g, 3.66 mmol) and sodium borohydride (0.383 g, 9.11 mmol) were placed in a two-necked round-bottomed flask. After replacement of air with argon gas in the flask, anhydrous isopropyl alcohol (25 mL) was added. The mixture was stirred and heated under reflux conditions for 1 h under argon atmosphere to generate sodium hydrogen selenide (NaHSe) in situ. A solution of **12a** (0.482 g, 1.28 mmol) in 1,4-dioxane (20 mL) was added to the resulting colorless solution. After further stirring under reflux conditions for 3 h, the reaction solution was cooled to room temperature and evaporated. To the residual material were added water (20 mL) and CH_2_Cl_2_ (20 mL), and extraction was conducted with CH_2_Cl_2_ (20 mL × 3). The combined organic layers were washed with brine (40 mL), dried over MgSO_4_, and concentrated under vacuum. The residual yellow solid was purified by silica gel column chromatography (EtOAc/*n*-hexane 1:4) and then by GPC to give a white solid of **13a**. Yield: 0.149 g, 49%; M.p. 120–122 °C; ^1^H-NMR (500 MHz, CDCl_3_): δ = 1.37 (s, 9H), 1.56 (s, 1H), 3.02–3.17 (m, 2H), 3.18–3.32 (m, 2H), 4.93 ppm (br s, 1H); ^13^C-NMR (125.8 MHz, CDCl_3_): δ= 24.8, 28.3, 49.5, 79.9, 153.8 ppm; ^77^Se-NMR (CDCl_3_): δ = 94.3 ppm; MS (APCI+): *m*/*z* calcd for C_8_H_16_NO_2_Se^+^: 238.03 [M + H]^+^; found: 238.04.

*(S)-3-(tert-Butoxycarbonylamino)tetrahydroselenopyran* (**13b**). A similar protocol to the synthesis of **13a** was applied, though EtOH was used as a solvent for generation of NaHSe and THF was used as a solvent to dissolve the starting material. Starting with **12b** (0.489 g, 1.30 mmol), **13b** was obtained as a white solid. Yield: 0. 235 g, 68%; M.p. 93–94 °C; ^1^H-NMR (500 MHz, CDCl_3_): δ = 1.38 (s, 9H), 1.42–1.49 (m, 1H), 1.66–1.70 (m, 1H), 1.83–1.90 (m, 1H), 2.36–2.58 (m, 3 H), 2.79–2.89 (m, 1H), 3.80 (m, 1H), 4.97 ppm (br s, 1H); ^13^C-NMR (125.8 MHz, CDCl_3_): δ = 19.1, 24.7, 26.0, 28.4, 32.7, 46.4, 49.4, 154.8 ppm; ^77^Se-NMR (CDCl_3_): δ = 115.8 ppm; MS (APCI+): *m*/*z* calcd for C_10_H_20_NO_2_Se^+^: 266.07 [M + H]^+^; found: 266.08.

*3-Aminoselenetane Hydrochloride* (**5**). HCl (4 mL, 5 M) was added to a solution of **13a** (0.14 g, 0.59 mmol) in Et_2_O (4 mL). The mixture was vigorously stirred for 18 h at 35 °C. After removal of the ether by evaporation, the resulting aqueous layer was diluted with water (50 mL) and lyophilized to give a white solid of **2** (99 mg, 97%). M.p. 172 °C (decomp); ^1^H-NMR (D_2_O): δ = 2.96–3.07 (m, 2H), 3.34–3.45 (m, 2H), 4.56–4.68 ppm (m, 1H); ^13^C-NMR (D_2_O) δ = 17.5, 48.6 ppm; ^77^Se-NMR (D_2_O): δ = 144.5 ppm; HRMS (APCI+): *m*/*z* calcd for C_3_H_8_NSe^+^: 137.9816 [M − Cl]^+^; found:137.9802.

*(S)-3-Aminotetrahydroselenopyran Hydrochloride* (**6**). A similar protocol to the synthesis of **5** was applied. Using **13b** (0.23 g, 0.89 mmol) as a starting material, **6** was obtained as a white solid. Yield: 0.16 g, 90%; M.p. 192 °C (decomp); ^1^H-NMR (D_2_O): δ = 1.41–1.51 (m, 1H), 1.79–1.89 (m, 1H), 1.91–1.95 (m, 1H), 2.20–2.26 (m, 1H), 2.46–2.61 (m, 2H), 2.63–2.76 (m, 2H), 3.42–3.54 ppm (m, 1H); ^13^C-NMR (D_2_O): δ = 18.7, 19.9, 26.5, 30.5 49.4 ppm; ^77^Se-NMR (D_2_O): δ = 136.2 ppm; HRMS (APCI+): *m*/*z* calcd for C_5_H_12_NSe^+^: 166.0129 [M − Cl]^+^; found: 166.0130.

### 3.2. Quantum Chemical Calculation

The Gaussian 09 software package (revision B.01) [44] was employed. The structures were optimized in water and in methanol at the B3LYP/6-31+G(d,p) level, using the polarizable continuum model (PCM). For the cyclic selenides **6** and **7**, all possible configurations were tested, and the global-energy-minimum structure was thus determined. Frequency calculations were performed for all the obtained structures to confirm that the structures had no imaginary frequencies. The energy-minimum structures were used for analyzing the HOMO energy levels and the intramolecular hydrogen-bond distances.

## 4. Conclusions

Here, we analyzed in-depth the GPx-like catalytic cycle of water-soluble amino-substituted cyclic selenides in water and in methanol on the basis of the component analysis of the reaction solution by means of ^77^Se-NMR and LC–MS spectroscopies. Under an aqueous condition at a physiological pH, it was revealed that two components (i.e., the selenide and the corresponding selenoxide), can mainly contribute to the antioxidative function though a slight contribution from the dihydroxy selenane **3a** was also suggested. Thus, cycle A in Scheme 1 should be a major cycle in water as reported in the literature [26,27,28,29]. In methanol, however, other active species, such as hydroxyselenonium **3** and hydroxy perhydroxy selenane **4** would be generated by over-reaction or over-oxidation of the corresponding selenoxide by an excess amount of H_2_O_2_. These species (i.e., **3** and **4**), should work as active oxidants against thiol substrates in consort with selenoxide **2** and **3a** if the velocities of reactions iii and/or vi in Scheme 1 are comparable or faster than that of reaction ii. This situation would be more feasible for amino-substituted cyclic selenides probably because the NH_3_^+^ group would transfer a proton to the selenoxide moiety to give hydroxyselenonium **3** in the absence of an additional proton source. Thus, a shift of the major catalytic cycle in methanol would make the GPx-like antioxidative function of selenides perplexing. The information obtained here will provide a valuable clue for design of novel selenide-based antioxidative enzyme mimics.

## Figures and Tables

**Table 1:** Summary of GPx-like catalytic activities of selenides in water and in MeOH along with the second-order rate constants for oxidation and the HOMO (highest occupied molecular orbital) energy levels.

Selenides	ν_0_ (μM·min^−1^) ^a^	*t*_50_ (min) ^b^	*k*_ox_ (M^−1^·s^−1^) ^c^	HOMO in Water (eV) ^d^	Substituents
No catalyst	32.2 (±3.2)	>300 >300 (4~10 equiv. HCl)	‒	‒	‒
**5**	61.3 (±3.4)	24	^f^	‒6.39	NH_3_^+^_ax_
**6**	52.6 (±2.1)	17 5 (+ 4 equiv. HCl) 3 (+10 equiv. HCl)	0.23 (± 0.02)	‒6.56 (ax)	NH_3_^+^_ax_
MAS^red^ (**7**) ^e^	59.8 (±1.1)	14	0.47 (± 0.05)	‒6.44	NH_3_^+^_ax_
DAS^red^ (**8**) ^e^	47.2 (±4.3)	7	0.14 (±0.02)	‒5.32	NH_3_^+^_ax_, NH_3_^+^_ax_
DHS^red^ (**9**) ^e^	54.2 (±4.0)	40	0.57 (± 0.03)	‒6.16	OH_ax_, OH_ax_

^a^ Initial velocities (ν_0_) of H_2_O_2_ reduction in phosphate buffer at pH 7.4 and 25 °C; ^b^ Reaction times for 50% conversion of DTT^red^ to DTT^ox^ in CD_3_OD estimated from Figure 3B; ^c^ The second-order rate constants for the reaction of selenide + H_2_O_2_ → selenoxide + H_2_O in water; ^d^ Calculated at B3LYP/6-31+G(d,p) in water using the polarizable continuum model (PCM); ^e^ Data were quoted from Reference [29]; ^f^ Not determined. MAS^red^: monoamino cyclic selenide; DAS^red^: diamino cyclic selenide; DHS^red^: dihydroxy cyclic selenide

## Supplementary Materials

Supplementary materials (Synthesis of mesylates **12a** and **12b**; ^1^H, ^13^C, and ^77^Se-NMR spectra of **5**, **6**, **13a** and **13b**; Figures S1‒S12; Quantum chemical calculations of the selenoxides corresponding **6** and **7**) are available online.
