# Supplementary material for: Glutathione Peroxidase-Like Activity of Amino-Substituted Water-Soluble Cyclic Selenides: A Shift of the Major Catalytic Cycle in Methanol
Source: Molecules. 2017 Feb 25;22(3):354. doi: 10.3390/molecules22030354 (PMC6155421; doi:10.3390/molecules22030354)
Supplement: Supplementary file 1 [file molecules-22-00354-s001.pdf]

Glutathione Peroxidase-Like Activity of Amino-Substituted Water-Soluble Cyclic Selenides: A Shift of the Major Catalytic Cycle in Methanol

Kenta Arai*, Ayako Tashiro, Yuui Osaka, and Michio Iwaoka*

Department of Chemistry, School of Science, Tokai University, Kitakaname, Hiratsuka-shi, Kanagawa 259-1292, Japan.

* Authors to whom correspondence should be addressed; E-Mail: k-arai4470@tokai-u.jp (KA), miwaoka@tokai.ac.jp (MI)

Tel.: +81-463-58-1211; Fax: +81-463-50-2094.

Supporting Information

Contents

1. Synthesis of mesylates 12a and 12b

2. NMR spectra

2.1: 3-(*tert*-Butoxycarbonylamino)selenetane (**13a**) (^{1}H, ^{13}C, and ^{77}Se NMR)

2.2: (*S*)-3-(*tert*-Butoxycarbonylamino)tetrahydroselenopyran (**13b**) (^{1}H, ^{13}C, and ^{77}Se NMR)

2.3: 3-Aminoselenetane Hydrochloride (**5**) (^{1}H, ^{13}C, and ^{77}Se NMR)

2.4: (*S*)-3-Aminotetrahydroselenopyran Hydrochloride (**6**) (^{1}H, ^{13}C, and ^{77}Se NMR)

3. Supplemental Figures

Figure S1: ^{77}Se NMR spectral changes during acidification and neutralization of the selenoxide derived from selenide **6** in D_2O.

Figure S2: ^{77}Se NMR spectral changes during redox reactions of **5** in D_2O at 298 K.

Figure S3: ^{77}Se NMR spectral changes during redox reactions of **7** in D_2O at 298 K.

Figure S4: ^{77}Se NMR spectral changes during acidification and neutralization of the selenoxide derived from selenide **7** in D_2O.

Figure S5: ^{77}Se NMR spectral changes during redox reactions of **9** in D_2O at 298 K.

Figure S6: ^{77}Se NMR spectral changes during acidification and neutralization of the selenoxide corresponding to selenide **9** in D_2O at 298 K.

Figure S7: LC-MS (ESI+) spectrum of the sample solution obtained when the selenoxide derived from selenide **6** was over-oxidized with 4 equivalents of H_2O_2 in water at 25 ℃ in the presence of HCl.

Figure S8: LC-MS (APCI+ and ESI+) spectral changes during over-oxidation of the selenoxide derived from selenide **7** in H_2O

Figure S9: ^{77}Se NMR spectral changes during acidification and neutralization of the selenoxide derived from selenide **7** in CD_3OD

Figure S10 LC-MS (APCI+) spectra changes during oxidation of the selenoxide derived from selenide **7** in MeOH at 25 ℃

Figure S11: Spectroscopic analyses during redox reactions of monoamino selenide **6** in methanol. (A) ^{77}Se NMR in CD_3OD and (B) LC-MS analysis in MeOH.

Figure S12: Spectroscopic analyses during redox reactions of selenide **9** in methanol. (A) ^{77}Se NMR in CD_3OD and (B) LC-MS analysis in MeOH.

4. Quantum chemical calculations of the selenoxide corresponding 6 and 7

1. Synthesis of mesylates 12a and 12b

The synthetic route for **12a** and **12b** are shown below (Scheme S1).

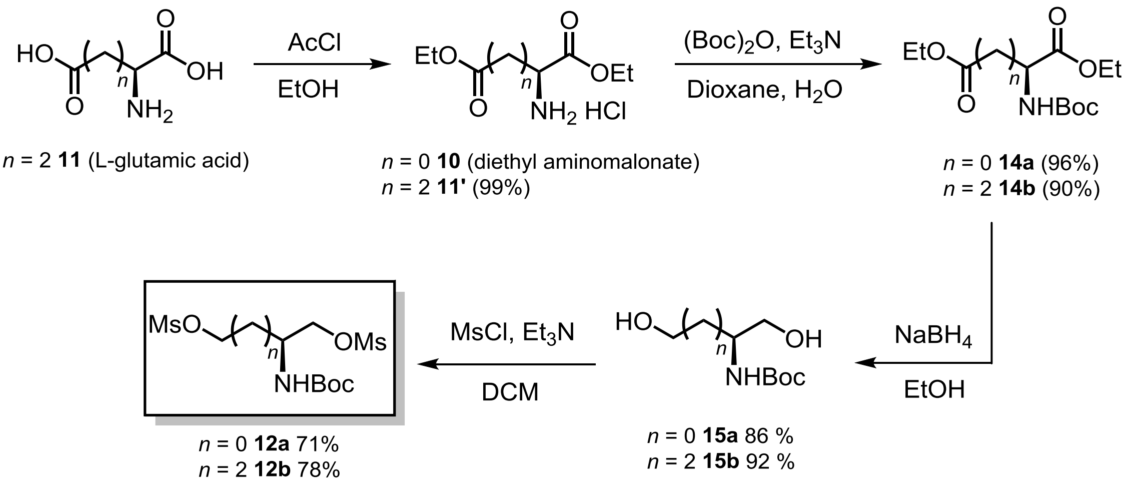

Scheme 1. Synthesis of **12a** and **12b**.

Diethyl L-glutamate hydrochloride (**11′**)

EtOH (42 mL) was placed in a round-bottomed flask and cooled to 0 ℃ in an ice bath. Acetyl chloride (3.6 mL, 50.0 mmol) was then slowly added to the EtOH with keeping the temperature and magnetically string. After the reaction solution was starred at 0 ℃ for 30 min, *L*-glutamic acid (3.68 g, 25.0 mmol) was added to the mixture solution. The reaction solution was stirred under a reflux condition for 4 h. The resulting solution was evaluated under vacuum to obtain a colorless oil of **11′**. Yield: 6.00 g, quant; R_f: 0.49 (EtOH/EtOAc 1:1);^{1}H NMR (500 MHz, CD_3OD): δ = 1.28 (t, *J*=7.2 Hz, 3H), 1.35 (t, *J*=7.2 Hz, 3H), 2.15–2.29 (m, 2H), 2.54–2.66 (m, 2H), 4.14 (t, *J*=6.7 Hz, 1H), 4.16–4.10 (m, 2 H), 4.30–4.35 ppm (m, 2H); ^{13}C NMR (125.8 MHz, CD_3OD): δ = 13.0, 13.1, 25.2, 29.0, 51.9, 60.6, 62.4, 168.7, 172.2 ppm.

Diethyl 2-((tert-butoxycarbonyl)amino)malonate (**14a**)

Et_3N (2.90 mL, 20.8 mmol) was added to a solution of diethyl 2-aminomalonate hydrochloride (**10**) (4.00 g, 18.9 mmol) in 1,4-dioxane:H_2O (5:2, 17 mL), and the solution was magnetically stirred on ice. A solution of Boc_2O (4.74 g, 21.7 mmol) in the same solvent (4 mL) was slowly added via a syringe, and the mixture solution was stirred at 0 ℃ for 15 min, and then at 55 ℃ for 15 h. The resulting yellow solution was concentrated to 10 mL under vacuum. The solution was added with water (40 mL), and the aqueous solution was extracted with Et_2O (30 mL × 3). The combined organic layers were washed with saturated aqueous solution of $NaHCO_3$ (40 mL × 2), water (40 mL × 2), and brine (40 mL × 1), dried over $MgSO_4$, and concentrated under vacuum to obtain a colorless oil of **14a**. Yield: 5.02 g, 96%; R_f: 0.71 (EtOAc/*n*-hexane 1:1); ^{1}H NMR (500 MHz, $CDCl_3$): δ = 1.25 (t, *J*=7.2 Hz, 6H) 1.41 (s, 9H), 4.16–4.16 (m, 4H), 4.9 (d, *J*=10.0 Hz, 1H), 5.60 ppm (br d, J=5.0 Hz, 1H); ^{13}C NMR (125.8 MHz, $CDCl_3$): δ = 13.9, 28.2, 62.4, 67.0, 80.5, 154.8, 166.6 ppm.

Diethyl (tert-butoxycarbonyl)-L-glutamate (**14b**)

A similar protocol to the synthesis of **14a** was applied. **11′** (5.92 g, 24.7 mmol) was used as the starting material. Et_3N (3.79 mL, 27.2 mmol) and Boc_2O (6.20 g, 27.2 mmol) were used as the reagents. **14b** was obtained as colorless oil. Yield: 6.75 g, 90%; R_f: 0.63 (Et_2O/*n*-hexane 1:1); ^{1}H NMR (500 MHz, $CDCl_3$): δ = 1.25 (t, *J*=7.1 Hz, 3H), 1.28 (t, *J*=7.1 Hz, 3H), 1.43 (s, 9H), 1.90–1.98 (m, 1H), 2.14–2.21 (m, 2H), 2.33–2.45 (m, 2H), 4.13 (q, *J*=7.1, 2 H), 4.19 (q, *J*=7.1, 2H), 4.25–4.35 (m, 1H),5.16 ppm (br d, 1H); ^{13}C NMR (125.8 MHz, $CDCl_3$): δ = 14.1, 14.2, 27.8, 28.3, 30.4, 53.0, 60.6, 61.5, 79.9, 155.4, 172.3, 172.8 ppm.

tert-Butyl (1,3-dihydroxypropan-2-yl)carbamate (**15a**)

90% sodium borohydride (4.21 g, 100.2 mmol) was slowly added to the solution of **14a** (2.77 g, 10.0 mmol) in dry EtOH (40 mL) on ice, the solution was magnetically stirred for 30 min at

0 ℃ and then under reflux condition for 1 h. The resulting white cake was pulverized by using a spatula and added with in brine (50 mL), and the mixture solution was vigorously stirred for 10 min at room temperature. After removing a suspended white material by filtration under reduced pressure, the obtained filtrate was concentrated in vacuo to 40 mL. The remaining aqueous solution was extracted with Et_2O (30 mL × 4). The combined organic layers were washed with brine (60 mL × 1), dried over $MgSO_4$, and concentrated under vacuum to obtain a white solid of **15a**. Yield: 1.65 g, 86%; R_f: 0.74 (EtOAc/*n*-hexane 5:1); ^{1}H NMR (500 MHz, $CDCl_3$): δ = 1.46 (s, 9H), 2.86 (br s, 2H), 3.68–3.80 (m, 5H), 5.43 ppm (br d, *J*=6.3 Hz, 1H); ^{13}C NMR (125.8 MHz, $CDCl_3$): δ = 28.4, 53.1, 62.8, 80.0, 156.5 ppm.

tert-Butyl (S)-(1,5-dihydroxypentan-2-yl)carbamate (**15b**)

A similar protocol to the synthesis of **15a** was applied. **14b** (3.03 g, 10.0 mmol) was used as the starting material. 90% sodium borohydride (4.20 g, 100.0 mmol) was used as the reagents. **15b** was obtained as colorless oil. Yield: 2.01 g, 92%; R_f: 0.71 (EtOAc/n-hexane 2:1); ^{1}H NMR (500 MHz, CD_3OD): δ = 1.46 (s, 9H), 1.55–1.70 (m, 4H), 3.47–3.52 (m, 3H), 3.58 (t, *J*=6.5 Hz, 2H), 5.31 (br s, 1H) ppm; ^{13}C NMR (125.8 MHz, CD_3OD): δ = 14.1, 27.4, 28.7, 51.2, 61.4, 64.1, 78.5, 157.0 ppm.

2-(tert-Butoxycarbonylamino)-propane-1,3-diyl dimethanesulfonate (**12a**)

Et_3N (1.15 mL, 8.22 mmol) was added to a solution of **15a** (1.34 mg, 7.00 mmol) in CH_2Cl_2 (40 mL) and the solution was stirred for 10 min and then cooled to 0 ℃. Methanesulfonyl chloride (2.17 mL, 28.0 mmol) was added over a period of 5 min, and the solution stirred at 0 ℃ for 30 min and then at room temperature for 16 h. Water was added, and the aqueous phase was extracted with CH_2Cl_2 (40 mL × 3). The combined organic phases were washed with saturated aqueous solution of $NaHCO_3$ (60 mL × 2), NH_4Cl (60 mL × 2), and brine (60 mL × 2), and dried over $MgSO_4$ and the concentrated under vacuum to give a yellow solid.

The obtained crude product was purified by silica gel column chromatography (EtOAc/CH_2CH_2 1:4) to give a white solid of **12a**. Yield: 1.73, 71%; *R*f: 0.63 (EtOAc/CH_2Cl_2 1:1); ^{1}H NMR (500 MHz, $CDCl_3$): δ = 1.47 (s, 9H), 3.15 (s, 6H), 4.25–4.28 (m, 1H), 4.31–4.41 (m, 4H), 5.04 (br s, J=10.0 Hz, 1H) ppm; ^{13}C NMR (125.8 MHz, $CDCl_3$): δ = 28.3, 37.5, 48.4, 66.8, 80.9, 155.0 ppm.

(S)-2-(tert-Butoxycarbonylamino)-pentane-1,5-diyl dimethanesulfonate (**12b**)

A similar protocol to the synthesis of **12a** was applied. **15b** (2.00 g, 9.12 mmol) was used as the starting material. Et_3N (4.5 mL, 31.9 mmol) and methanesulfonyl chloride (1.8 mL, 22.8 mmol) were used as the reagents. **12b** was obtained as a white solid. Yield: 2.65 g, 78%; *R*f: 0.60 (EtOAc/CH_2Cl_2 1:1); ^{1}H NMR (500 MHz, $CDCl_3$): δ = 1.46 (s, 9H), 1.57–1.98 (m, 4H), 3.04 (s, 3H), 3.06 (s, 3H), 3.91–3.93 (m, 1H), 4.21–4.30 (m, 4H), 4.74 ppm (br s, 1H); ^{13}C NMR (125.8 MHz, $CDCl_3$): δ = 25.7, 27.5, 28.3, 37.4, 49.1, 69.2, 71.0, 80.1, 154.8 ppm.

2. NMR spectra

2.1: 3-(*tert*-Butoxycarbonylamino)selenetane (**13a**)

^{1}H NMR in $CDCl_3$

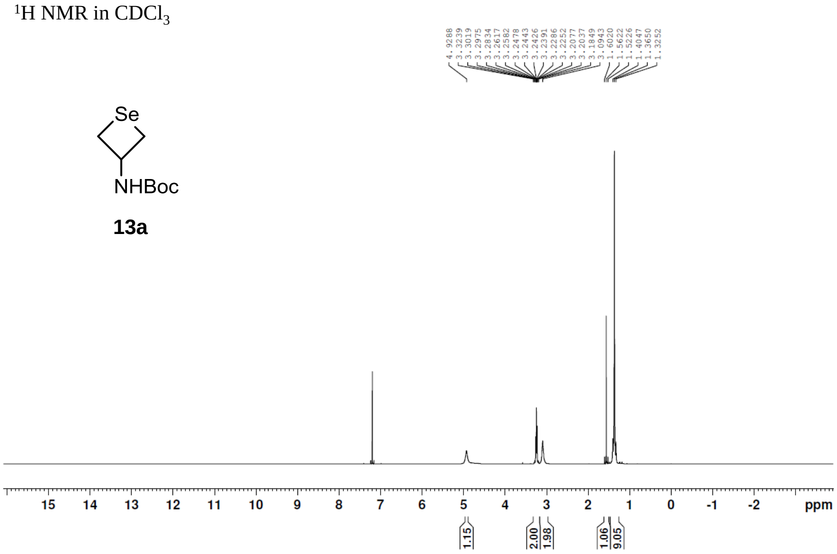

^{13}C NMR in $CDCl_3$

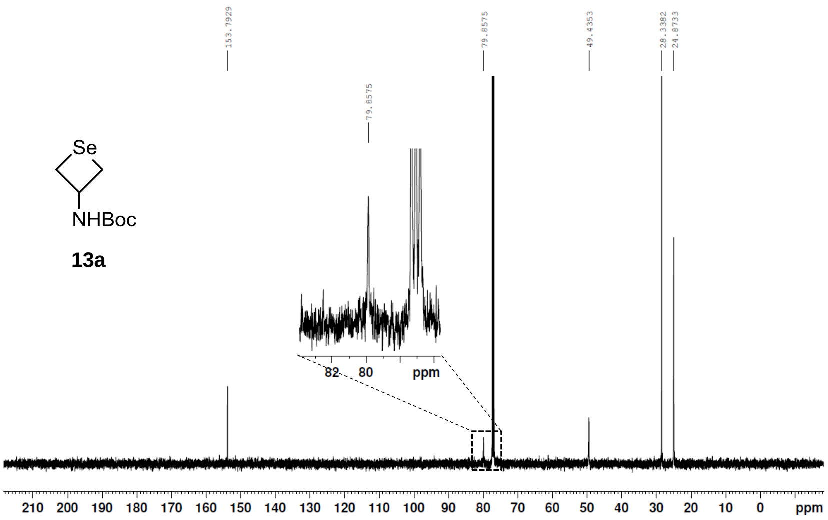

^{77}Se NMR in $CDCl_3$

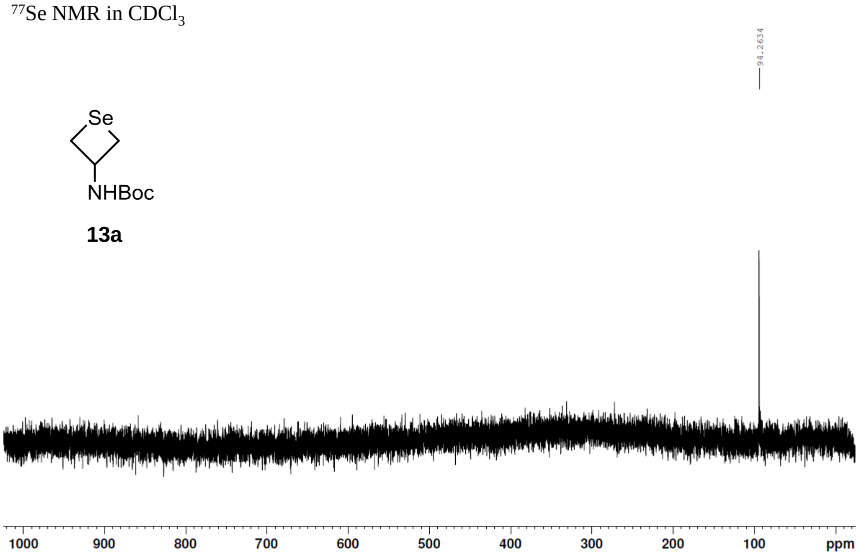

2.2: (*S*)-3-(*tert*-Butoxycarbonylamino)tetrahydroselenopyran (**13b**)

^{1}H NMR in $CDCl_3$

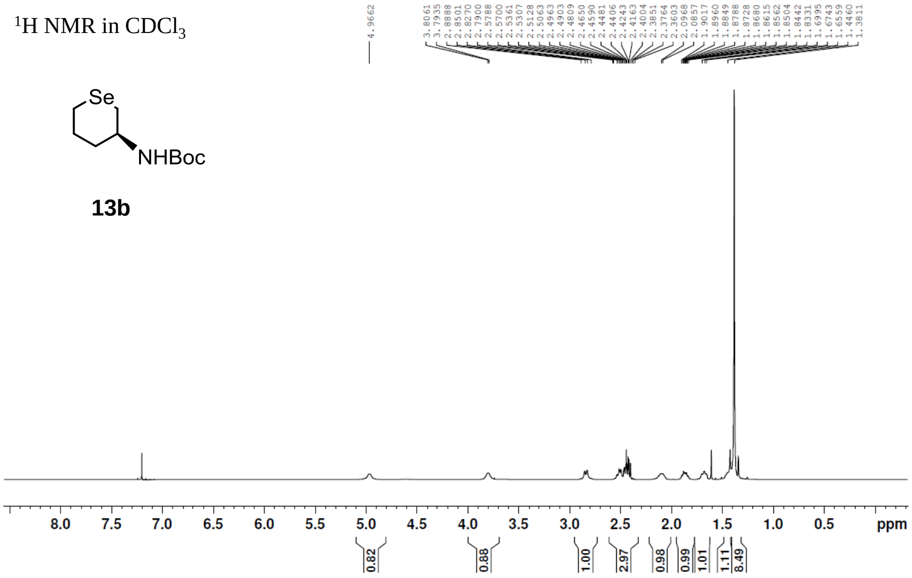

^{13}C NMR in $CDCl_3$

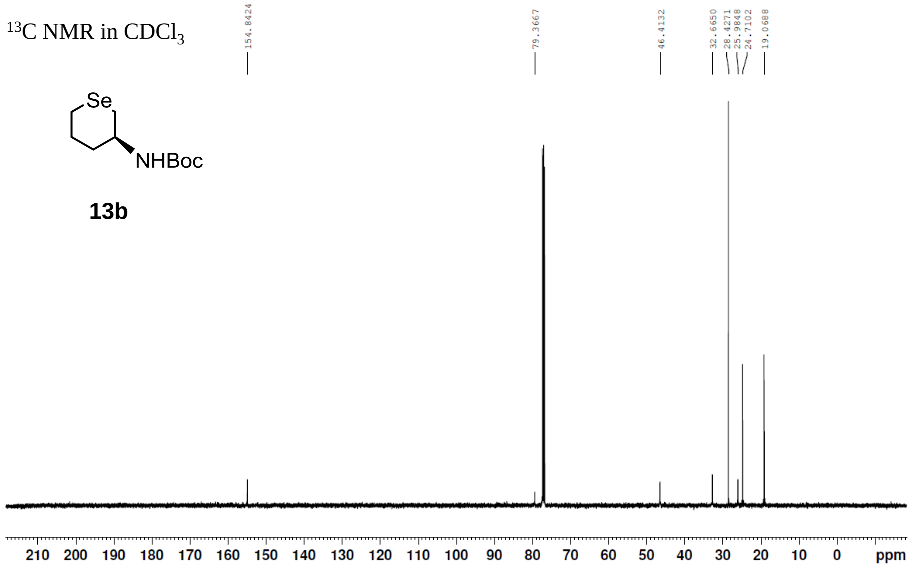

^{77}Se NMR in $CDCl_3$

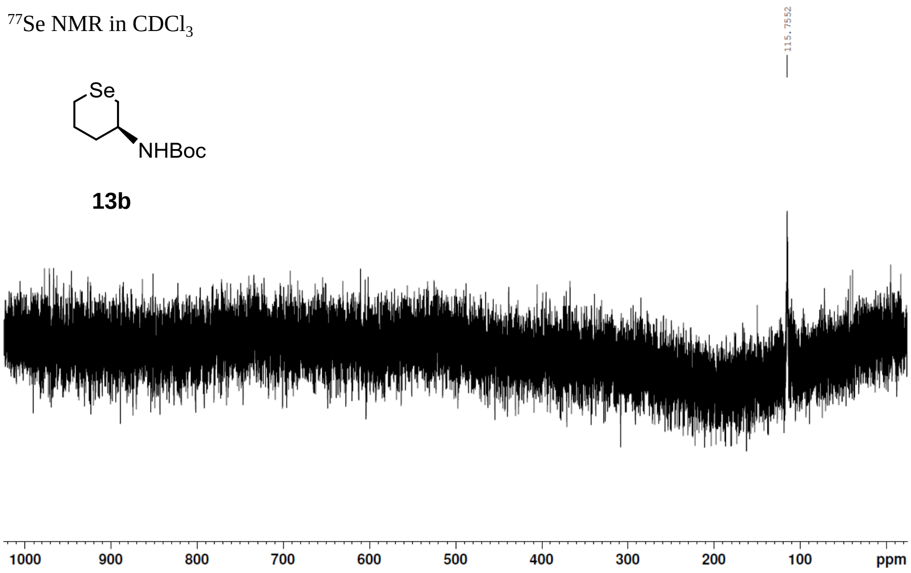

2.3: 3-Aminoselenetane Hydrochloride (**5**)

^{1}H NMR in D_2O

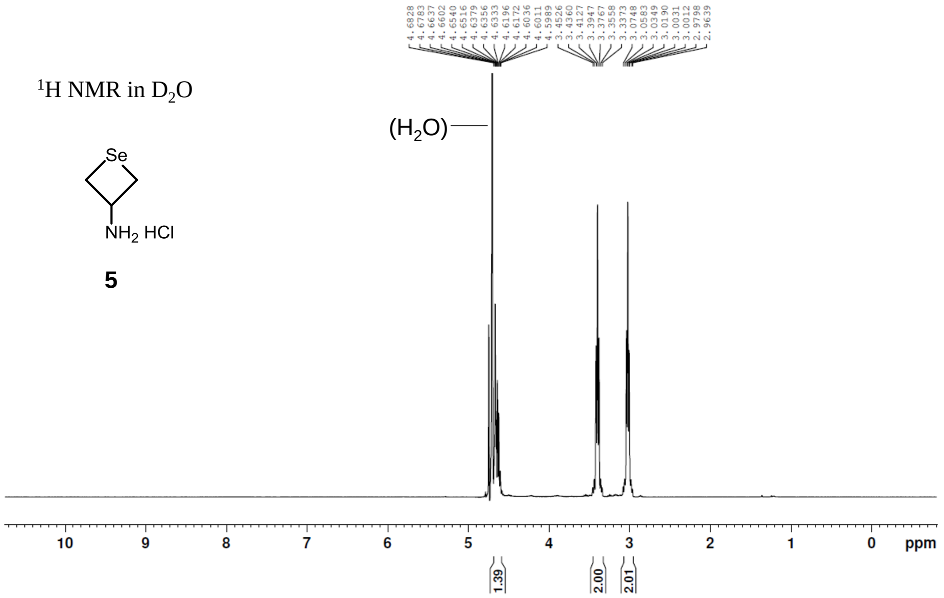

^{13}C NMR in D_2O

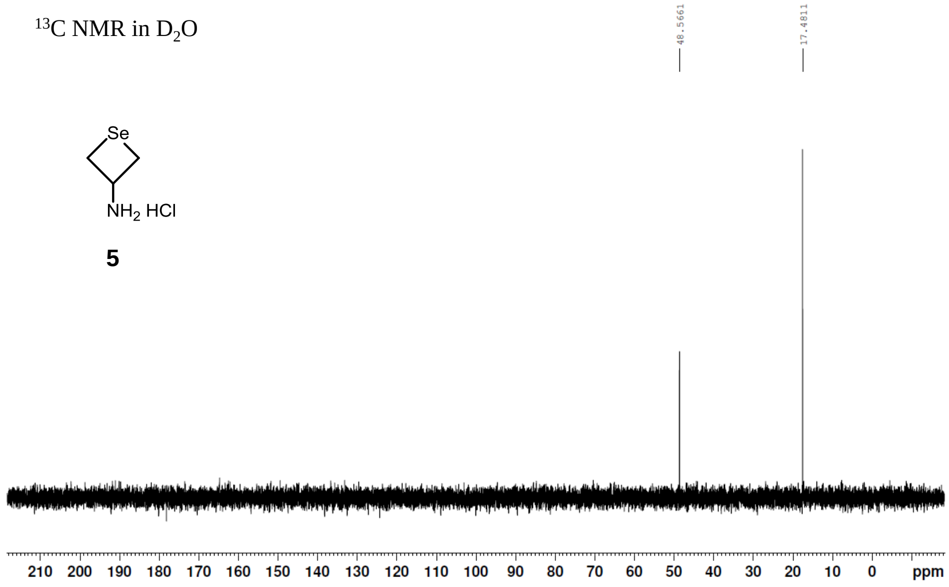

^{77}Se NMR in D_2O

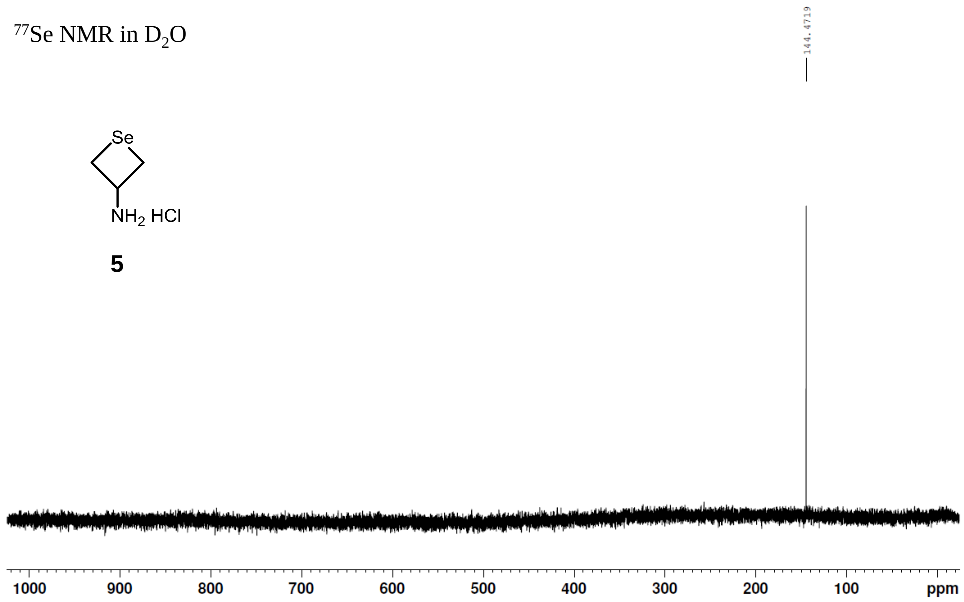

2.4: (*S*)-3-Aminotetrahydroselenopyran Hydrochloride (**6**)

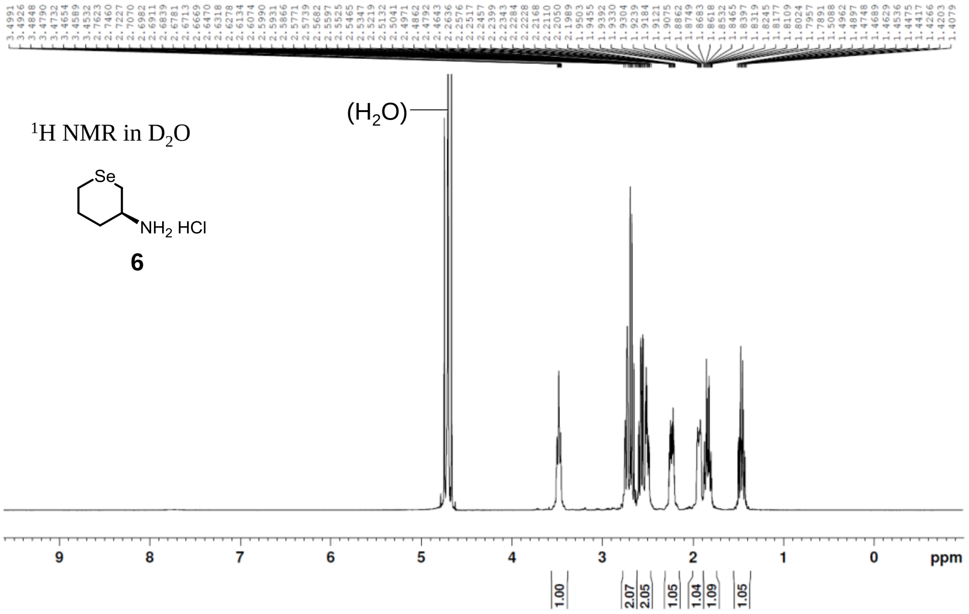

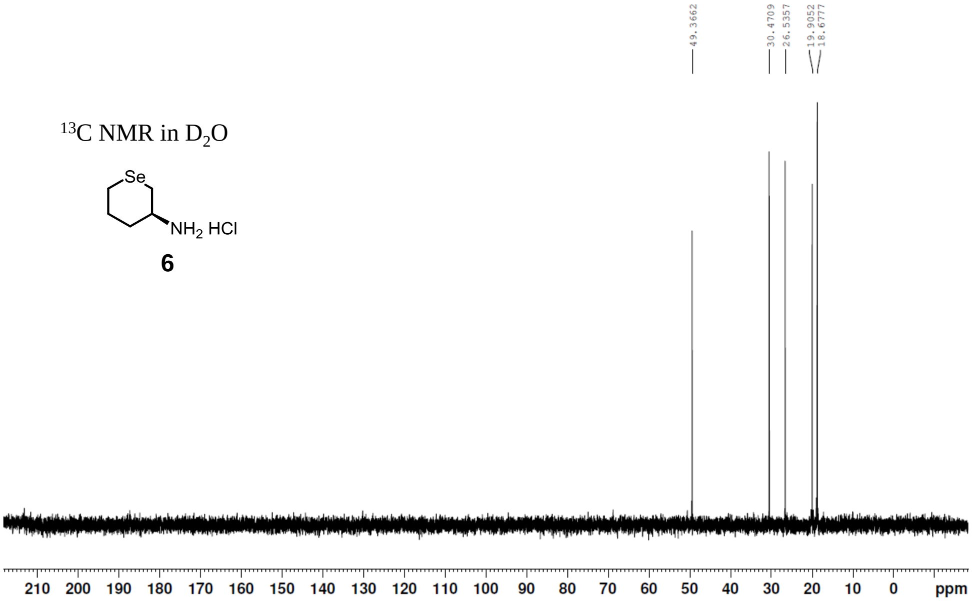

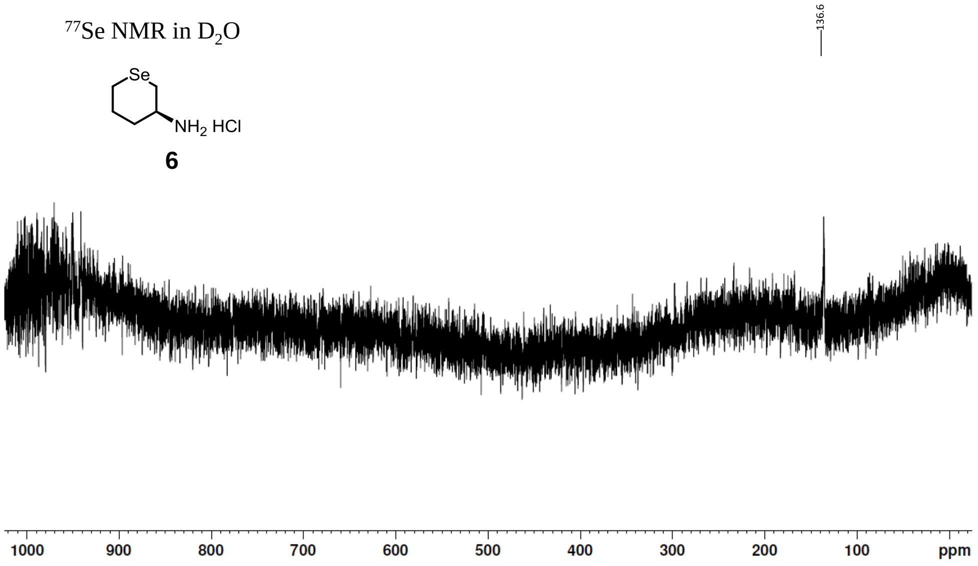

3. Supplemental Figures

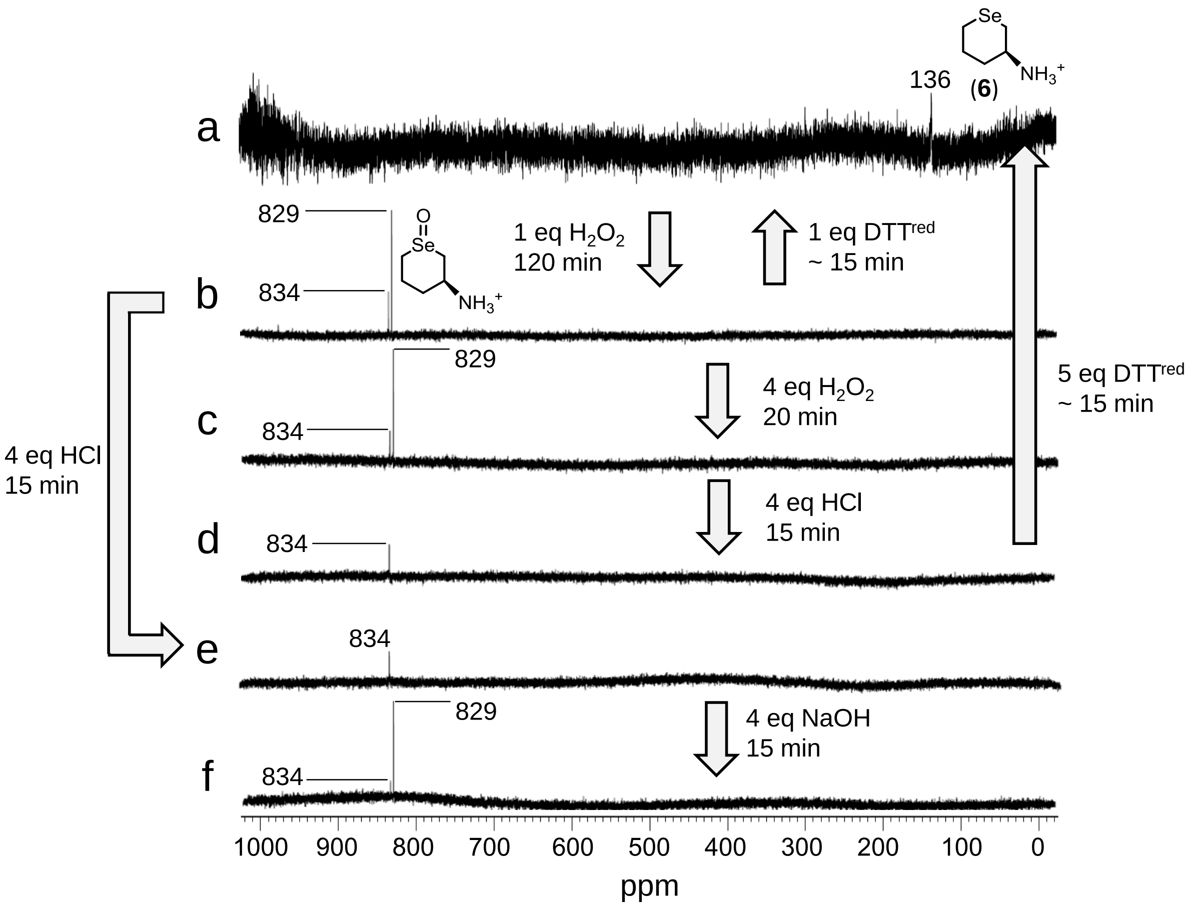

Figure S1: ^{77}Se NMR spectral changes during redox reactions of **6** and acidification and neutralization of the selenoxide derived from selenide **6** in D_2O at 298 K. Reaction conditions: **a**, Selenide **6** (0.024 mmol) in D_2O (500 μL). **b**, To **a** was added H_2O_2 (0.024 mmol). **c**, To **b** was added H_2O_2 (0.096 mmol). **d**, To **c** was added HCl (0.096 mmol). **e**, To **b** was added HCl (0.096 mmol). **f**, To **e** was added NaOH (0.96 mmol).

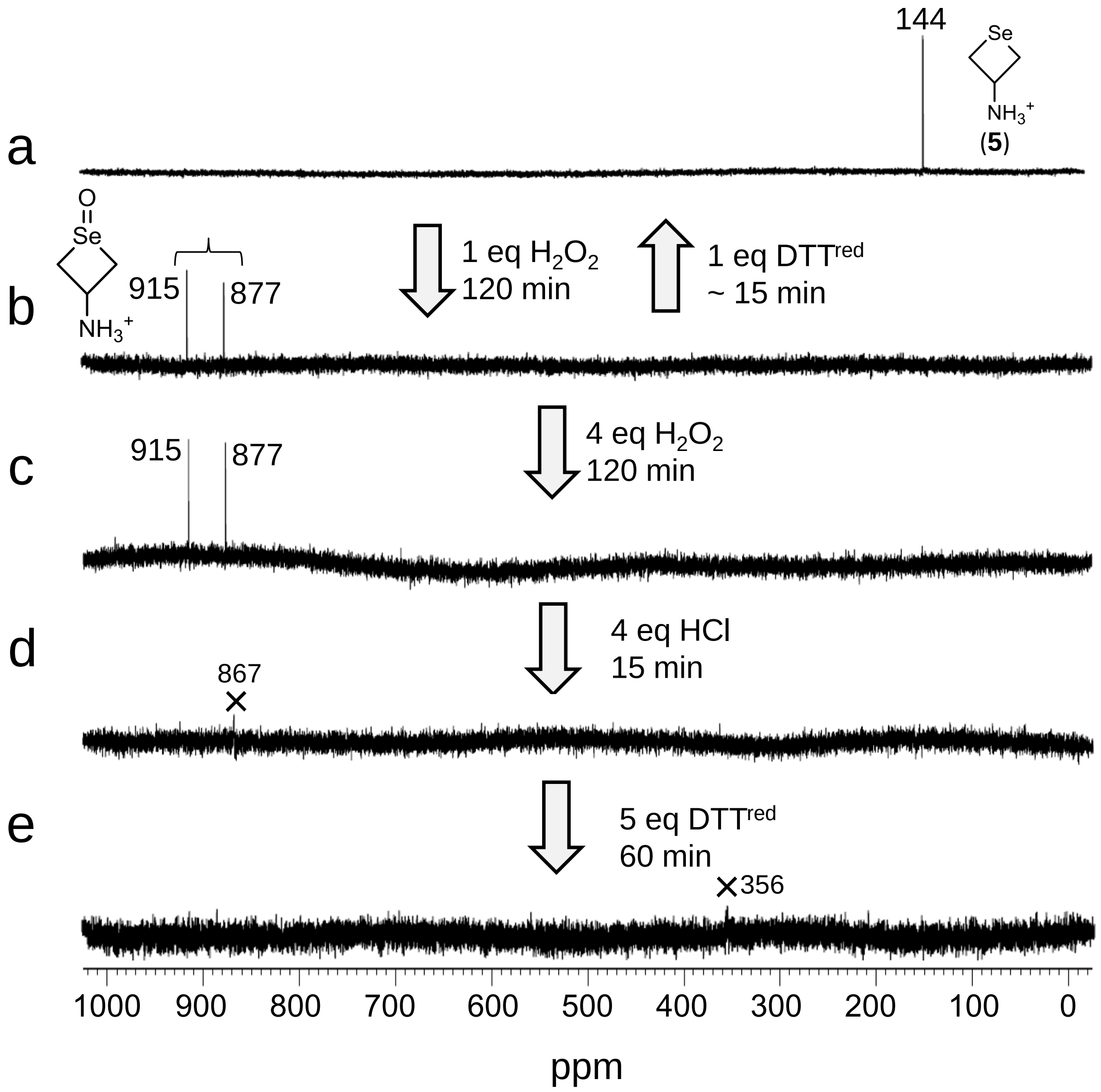

Figure S2: ^{77}Se NMR spectral changes during the redox reactions of **5** in D_2O at 298 K. Reaction conditions: **a**, Selenide **5** (0.024 mmol) in D_2O (500 μL). **b**, To **a** was added H_2O_2 (0.024 mmol). **c**, To **b** was added H_2O_2 (0.096 mmol). **d,** To **c** was added HCl (0.096 mmol). **e**, To **d** was added DTT^{red} (0.12 mmol).

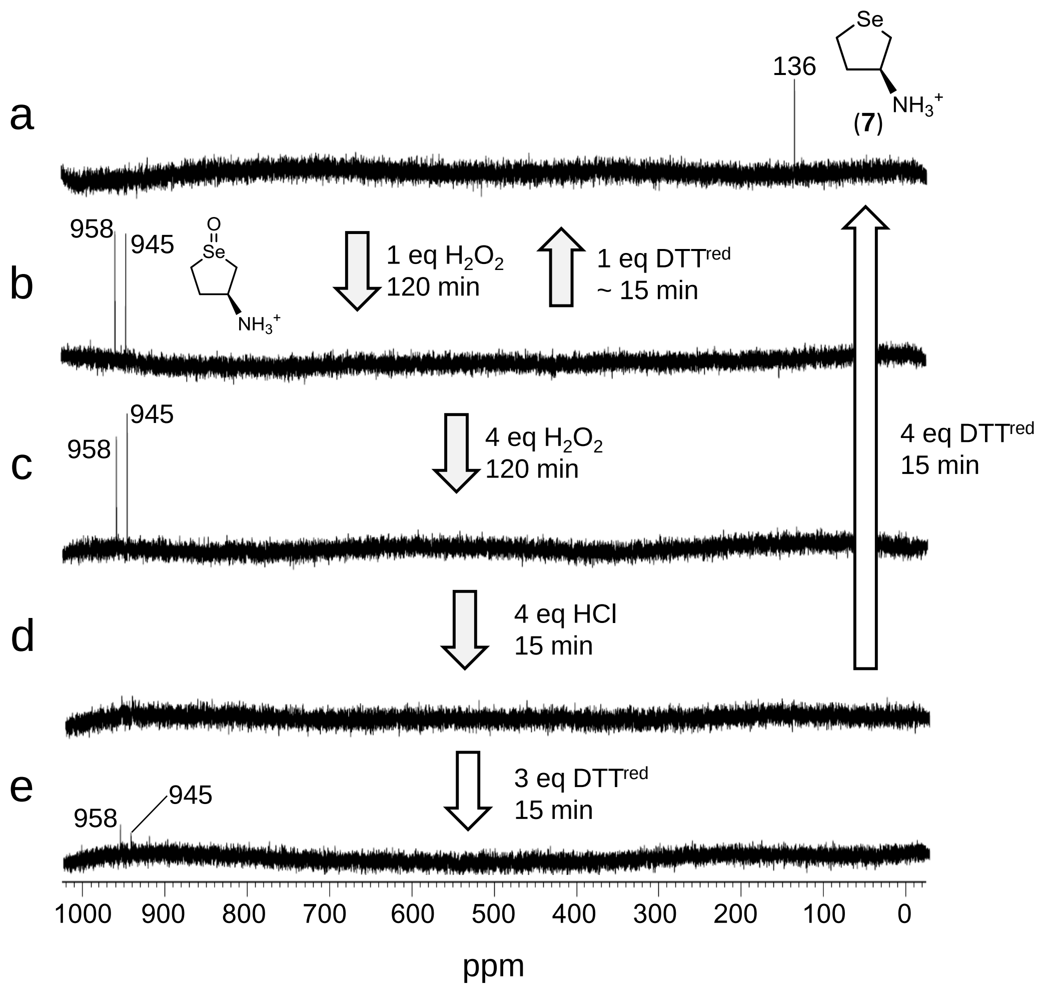

Figure S3: ^{77}Se NMR spectral changes during the redox reactions of **7** in D_2O at 298 K. Reaction conditions: **a**, Selenide **7** (0.024 mmol) in D_2O (500 μL). **b**, To **a** was added H_2O_2 (0.024 mmol). **c**, To **b** was added H_2O_2 (0.096 mmol). **d,** To **c** was added HCl (0.096 mmol). **e**, To **d** was added DTT^{red} (0.072 mmol).

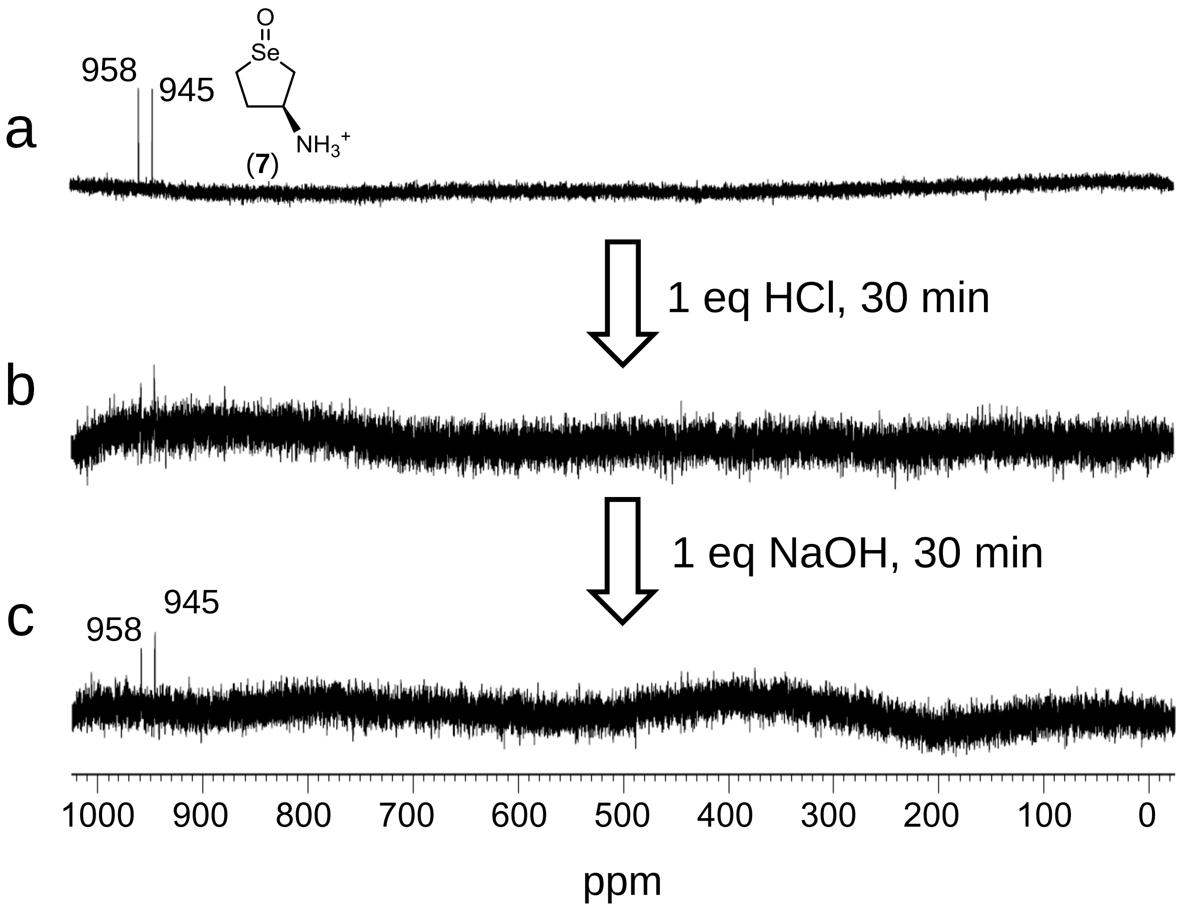

Figure S4: ^{77}Se NMR spectral changes during acidification and neutralization of the selenoxide derived from selenide **7** in D_2O at 298 K. Reaction conditions: **a**, Selenide **7** (0.024 mmol) and H_2O_2 (0.024 mmol) were mixed in D_2O (500 μL). **b**, To **a** was added HCl (0.024 mmol). **c**, To **b** was added NaOH (0.024 mmol).

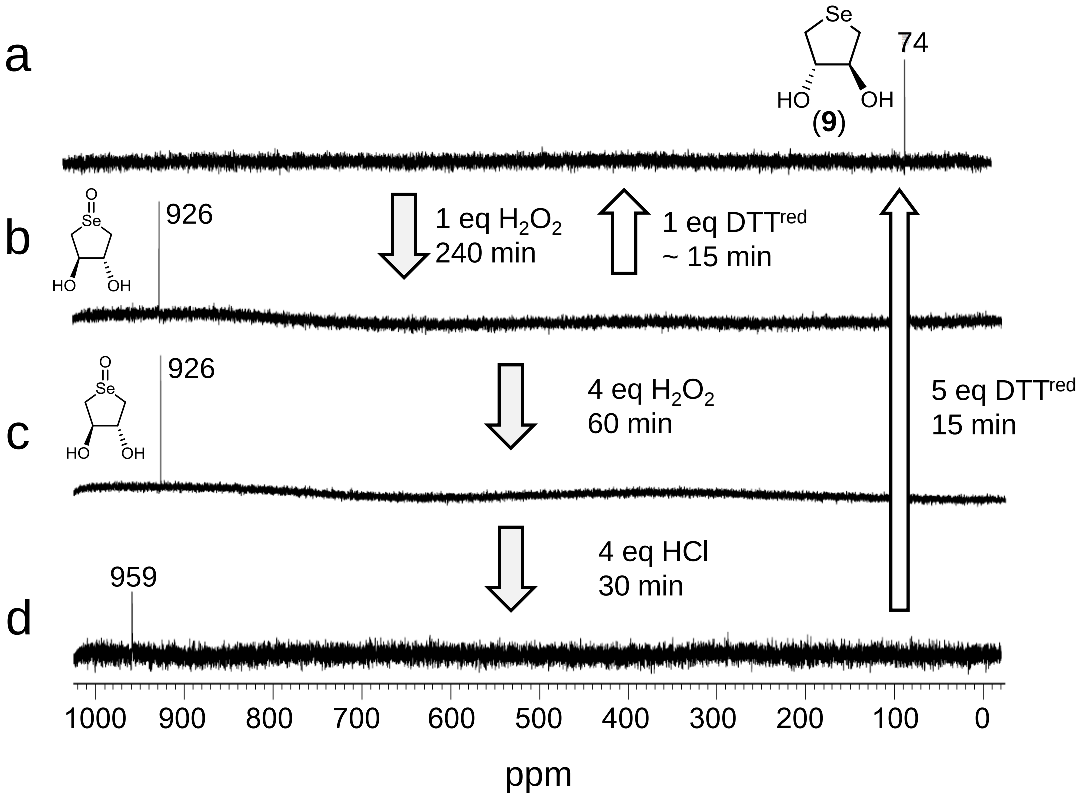

Figure S5: ^{77}Se NMR spectral changes during redox reaction of **9** in D_2O at 298 K. Reaction conditions: **a**, Selenide **9** (0.024 mmol) in D_2O (500 µL). **b**, To **a** was added H_2O_2 (0.024 mmol). **c**, To **b** was added H_2O_2 (0.096 mmol). **d,** To **c** was added HCl (0.096 mmol).

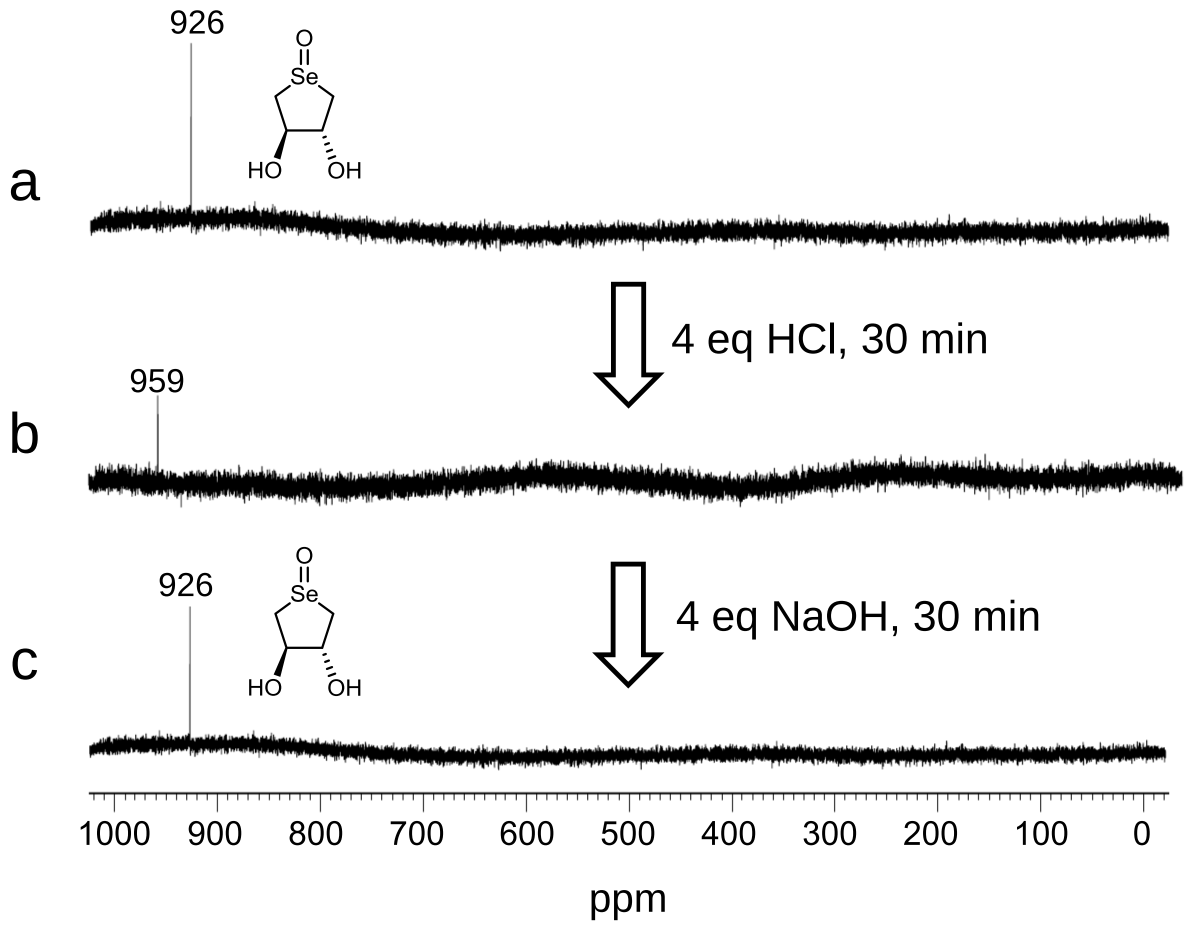

Figure S6: ^{77}Se NMR spectral changes during acidification and neutralization of the selenoxide derived from selenide **9** in D_2O at 298 K. Reaction conditions: **a**, Selenide **9** (0.024 mmol) and H_2O_2 (0.024 mmol) were mixed in D_2O (500 μL). **b**, To **a** was added HCl (0.096 mmol). **c**, To **b** was added NaOH (0.096 mmol).

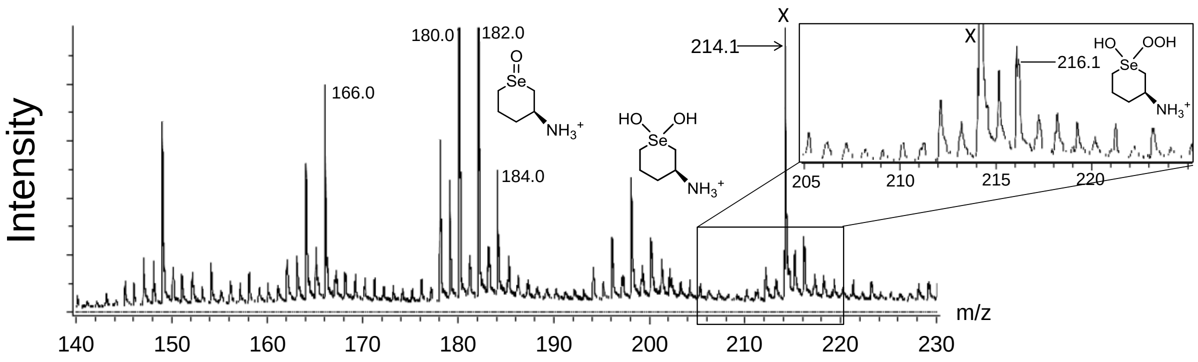

Figure S7: LC-MS (ESI+) spectrum of the sample solution obtained when the selenoxide derived from selenide **6** was over-oxidized with excess amounts of H_2O_2 in water at 25 ℃ in the presence of HCl. Selenide **6** (0.024 mmol) and H_2O_2 (0.12 mmol) was mixed in water (500 μL), and the resulting solution was incubated 18 h at 25 ℃ and added with HCl (0.096 mmol). The sample solution was directly injected into an ESI(+)-MS chamber from a syringe pump under a continuous flow at 30 μL/min.

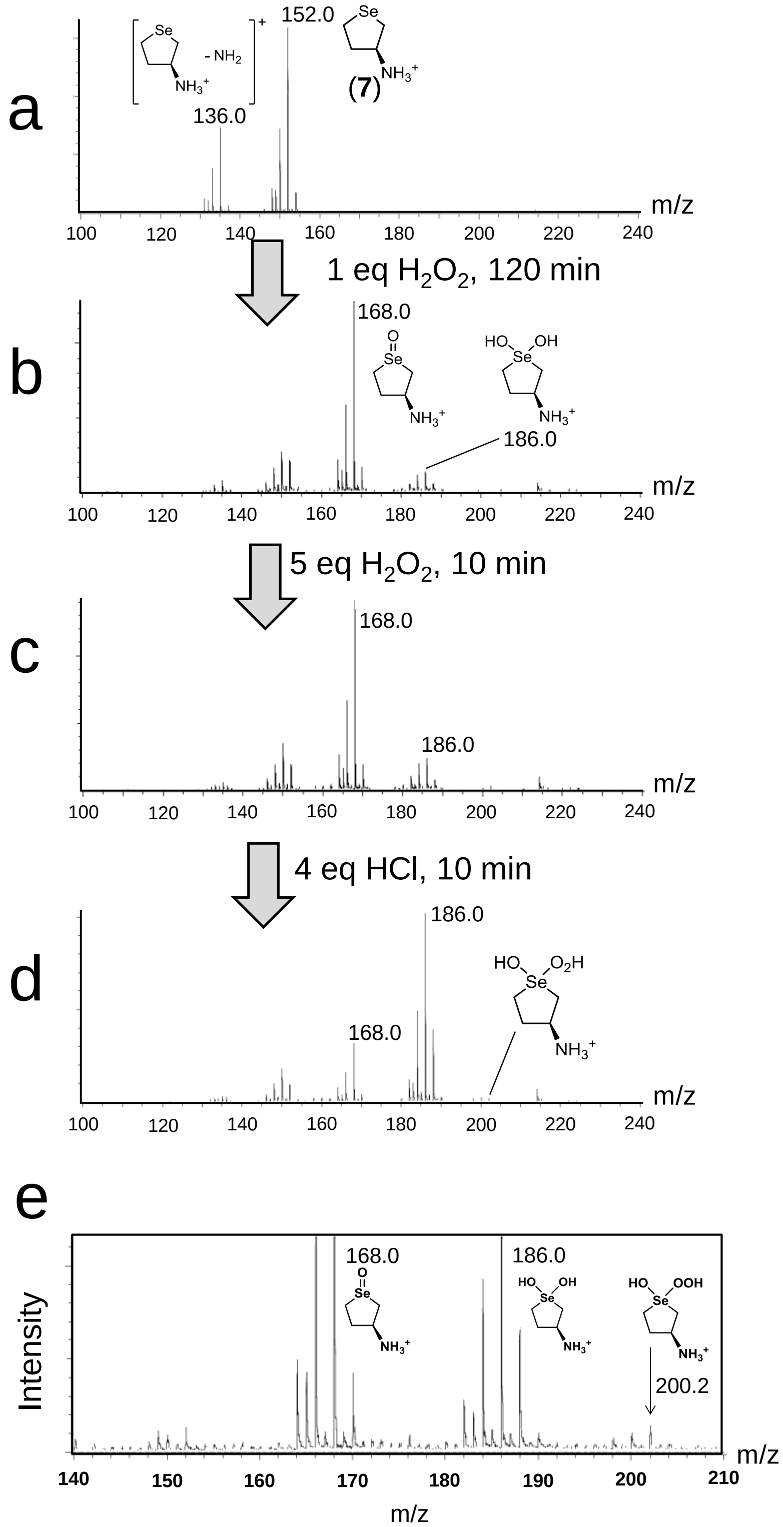

Figure S8: LC-MS (APCI+ and ESI+) spectra changes during oxidation of the selenoxide derived from selenide **7** in H_2O at 25 ℃. For **a**–**d**, H_2O (100%) was used as an eluent for the LC under a continuous flow at 0.3 mL/min, and 3 μL of the sample solution was injected into the LC and analyzed by APCI+ mode. For (**e**), the sample solution was directly injected into an ESI(+)-MS chamber from a syringe pump under a continuous flow at 30 μL/min. Reaction

conditions: **a**, Selenide **7** (0.038 mmol) in H_2O (800 μL). **b**, To **a** was added H_2O_2 (0.038 mmol). **c**, To **b** was added H_2O_2 (0.19 mmol). **d** and **e**, To **c** was added HCl (0.015 mmol)

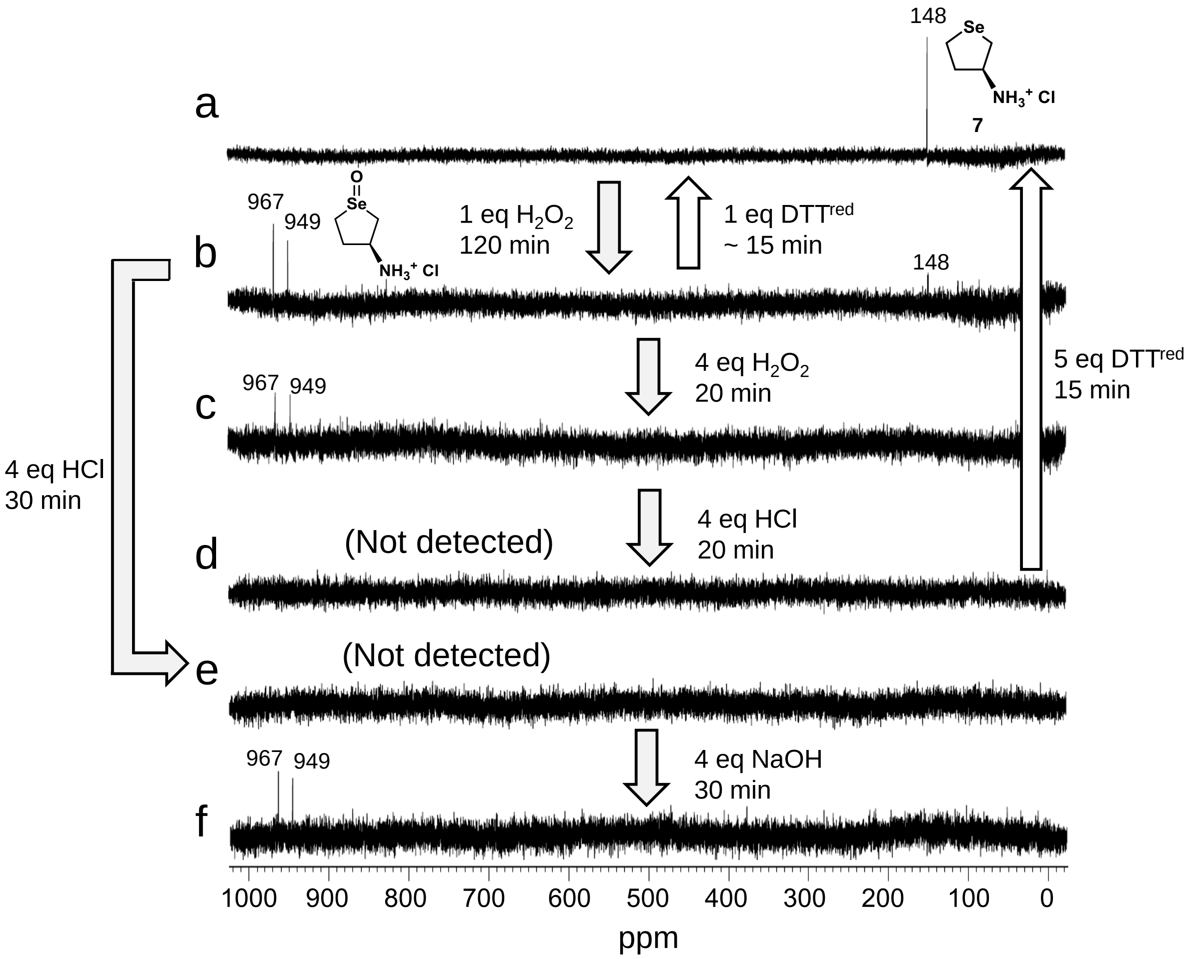

Figure S9: ^{77}Se NMR spectral changes during redox reactions of **7** and acidification and neutralization of the selenoxide derived from selenide **7** in CD_3OD at 298 K. Reaction conditions: **a**, Selenide **7** (0.024 mmol) in CD_3OD (500 μL). **b**, To **a** was added H_2O_2 (0.024 mmol). **c**, To **b** was added H_2O_2 (0.096 mmol). **d**, To **c** was added HCl (0.096 mmol). **e**, To **b** was added HCl (0.096 mmol). **f**, To **e** was added NaOH (0.096 mmol).

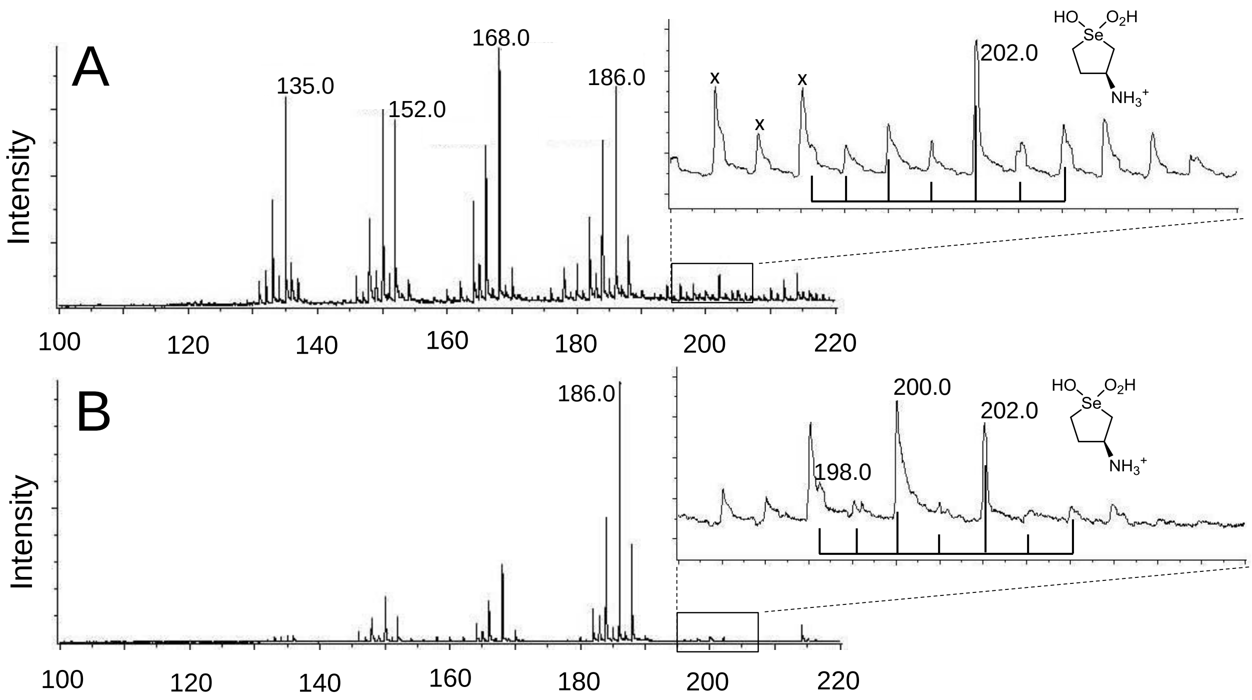

Figure S10: LC-MS (APCI+) spectra changes during oxidation of selenide **7** in MeOH at 25 ℃. MeOH (100%) was used as an eluent for the LC. Reaction conditions: (A) Selenide **7** (0.038 mmol) in MeOH was reacted with H_2O_2 (0.19 mmol) for 30 min. (B) Selenide **7** (0.038 mmol) in MeOH was reacted with H_2O_2 (0.19 mmol) in the presence of HCl (0.15 mmol) for 30 min.

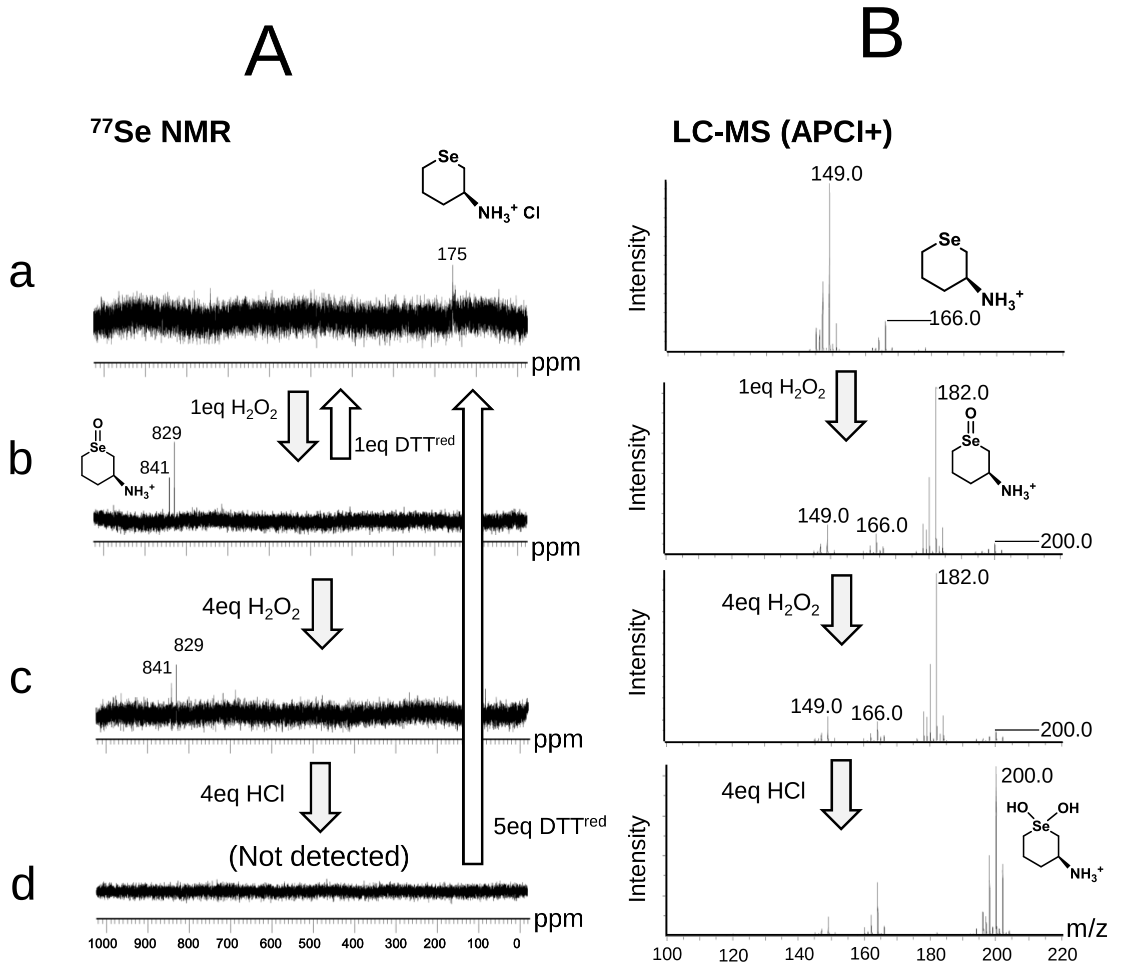

Figure S11: Spectroscopic analyses during redox reactions of monoamino selenide **6** in methanol. (A) ^{77}Se NMR spectral changes in CD_3OD at 297 K. Reaction conditions: **a**, Selenide **6** (0.024 mmol) in CD_3OD (500 μL). (**b**) To **a** was added H_2O_2 (0.024 mmol). (**c**) To **b** was added H_2O_2 (0.096 mmol). (**d**) To **c** was added HCl (0.096 mmol). (B) LC-MS spectra (APCI+) changes in MeOH at 25 ℃. MeOH (100%) was used as an eluent for the LC. Reaction conditions: **a**, Selenide **7** (0.038 mmol) in MeOH (800 μL). **b**, To **a** was added H_2O_2 (0.038 mmol). **c**, To **b** was added H_2O_2 (0.15 mmol). **d**, To **c** was added HCl (0.15 mmol)

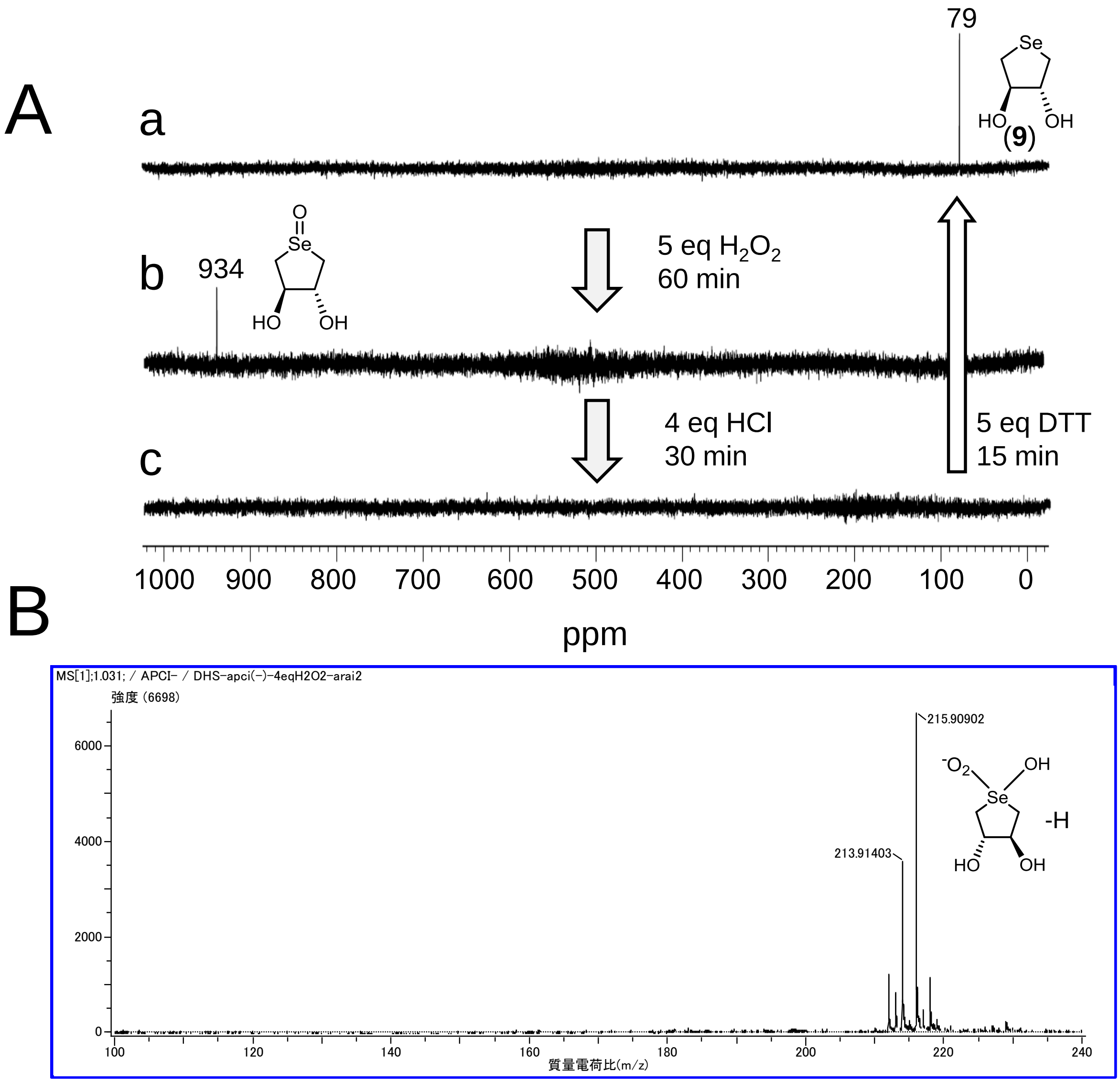

Figure S12: Spectroscopic analysis among the redox reactions of selenide **9** in methanol. (A) ^{77}Se NMR spectral changes in CD_3OD at 297 K. Reaction conditions: **a**, Selenide **9** (0.024 mmol) in CD_3OD (500 µL). (**b**) To **a** was added H_2O_2 (0.012 mmol). (**c**) To **b** was added HCl (0.096 mmol). (B) LC-MS (APCI–) analysis of hydroxy perhydroxy selenane **4** derived from **9.** The sample was prepared by mixing **9** (0.038 mmol) and H_2O_2 (0.152 mmol) in MeOH in the presence of HCl (0.152 mmol).

4. Quantum chemical calculations of the selenoxide corresponding 6 and 7

The selenodixe of **6** obtained in water with geometry optimization.

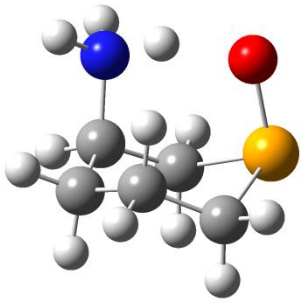

Calculation level: RB3LYP/PCM(water)/6-31+G(d,p)

E(RB3LYP): -2727.00248851 a.u.

Minimum frequency: 134.1 cm^{-1}

Atomic coordinates:

Center Number	Atomic Number	Atomic Type	Coordinates (Angstroms) X	Y	Z
1	6	0	-1.905409	0.957024	0.256940
2	6	0	-0.861152	1.671249	-0.618041
3	6	0	0.541392	1.693074	-0.019373
4	6	0	-0.281317	-0.769823	1.271746
5	6	0	-1.623384	-0.519410	0.577970
6	1	0	1.262058	2.205971	-0.659596
7	1	0	-0.816731	1.224635	-1.617490
8	1	0	-1.182277	2.708666	-0.762006
9	1	0	-1.992947	1.472540	1.220064
10	1	0	-2.889612	1.027768	-0.218322
11	1	0	-0.101666	-1.829561	1.466700
12	1	0	-0.230036	-0.226613	2.218322
13	1	0	-2.423028	-0.895219	1.221708
14	1	0	0.580725	2.128447	0.984386
15	34	0	1.244651	-0.149297	0.171293
16	1	0	-1.753908	-2.352113	-0.470201
17	1	0	-2.430539	-1.102806	-1.299234
18	1	0	-0.711389	-1.219838	-1.191070
19	7	0	-1.651818	-1.358362	-0.688080
20	8	0	0.839146	-0.843741	-1.339516

The selenodixe of **6** obtained in methanol with geometry optimization.

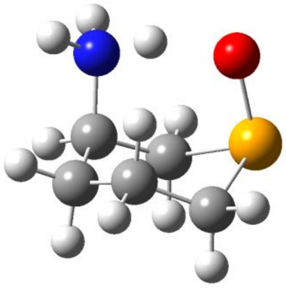

Calculation level: RB3LYP/PCM(methanol)/6-31+G(d,p)

E(RB3LYP): -2727.00063127 a.u.

Minimum frequency: 136.0 cm^{-1}

Atomic coordinates:

Center Number	Atomic Number	Atomic Type	Coordinates (Angstroms) X	Y	Z
1	6	0	-1.905611	0.956686	0.256279
2	6	0	-0.861036	1.670654	-0.618585
3	6	0	0.541072	1.693290	-0.018818
4	6	0	-0.281069	-0.768544	1.272723
5	6	0	-1.623182	-0.519524	0.578246
6	1	0	1.261538	2.206845	-0.658733
7	1	0	-0.815229	1.223109	-1.617530
8	1	0	-1.182538	2.707751	-0.763770
9	1	0	-1.993613	1.472962	1.218979
10	1	0	-2.889827	1.027201	-0.219009
11	1	0	-0.101030	-1.827863	1.469879
12	1	0	-0.230534	-0.223478	2.218293
13	1	0	-2.423002	-0.895443	1.221788
14	1	0	0.579628	2.128164	0.985249
15	34	0	1.244627	-0.149256	0.170838
16	1	0	-1.752606	-2.352647	-0.469273
17	1	0	-2.427175	-1.103227	-1.300470
18	1	0	-0.707282	-1.220054	-1.188857
19	7	0	-1.649931	-1.359084	-0.687560
20	8	0	0.836604	-0.843799	-1.338901

The selenodixe of **7** obtained in water with geometry optimization.

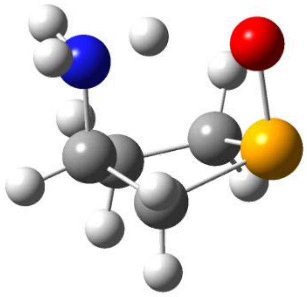

Calculation level: RB3LYP/PCM(water)/6-31+G(d,p)

E(RB3LYP): -2687.67971351 a.u.

Minimum frequency: 119.1 cm^{-1}

Atomic coordinates:

Center Number	Atomic Number	Atomic Type	Coordinates (Angstroms) X	Y	Z
1	6	0	0.009990	1.413491	-0.782626
2	6	0	0.421832	-0.258613	1.381426
3	6	0	1.645299	-0.040607	0.492746
4	6	0	1.448690	1.293642	-0.239991
5	1	0	-0.092000	1.129039	-1.831935
6	1	0	-0.426098	2.401162	-0.627046
7	1	0	0.385897	0.477236	2.187624
8	1	0	0.315292	-1.263601	1.793233
9	1	0	2.180693	1.421996	-1.043410
10	1	0	1.635758	2.083611	0.491810
11	8	0	-0.909140	-1.264506	-0.893956
12	34	0	-1.108666	0.066359	0.164995
13	1	0	2.571707	-0.039906	1.070264
14	1	0	2.374656	-0.997561	-1.256030
15	1	0	2.052300	-2.049008	-0.031999
16	1	0	0.740974	-1.379675	-0.876703
17	7	0	1.724817	-1.195849	-0.491901

The selenodixe of **7** obtained in methanol with geometry optimization.

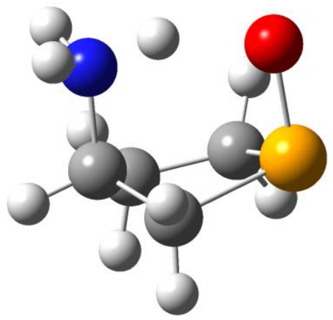

Calculation level: RB3LYP/PCM(methanol)/6-31+G(d,p)

E(RB3LYP): -2687.67776083 a.u.

Minimum frequency: 125.5 cm^{-1}

Atomic coordinates:

Center Number	Atomic Number	Atomic Type	Coordinates (Angstroms) X	Y	Z
1	6	0	0.010340	1.414438	-0.781630
2	6	0	0.421438	-0.257920	1.382277
3	6	0	1.644823	-0.041169	0.492892
4	6	0	1.449351	1.293068	-0.240306
5	1	0	-0.092562	1.130717	-1.831065
6	1	0	-0.425061	2.402376	-0.625460
7	1	0	0.385662	0.477760	2.188667
8	1	0	0.314559	-1.262997	1.793894
9	1	0	2.180587	1.419757	-1.044729
10	1	0	1.639087	2.083367	0.490472
11	8	0	-0.905134	-1.264097	-0.893913
12	34	0	-1.108487	0.066334	0.164597
13	1	0	2.571663	-0.041218	1.069894
14	1	0	2.371420	-1.000117	-1.256112
15	1	0	2.045738	-2.050725	-0.031792
16	1	0	0.734035	-1.377043	-0.875953
17	7	0	1.721254	-1.196424	-0.491747
